# Inhibition of ferroptosis by microbiota‐derived lithocholic acid underlies the intestinal radioprotection of a host defensin‐derived oligopeptide

**DOI:** 10.1002/imt2.70113

**Published:** 2026-02-19

**Authors:** Xue Ouyang, Yingjuan He, Zihao Jin, Shaobo Wang, Gaomei Zhao, Xiaona Su, Jia Du, Yin Chen, Chengzhang Tan, Xin Li, Songling Han, Bin Wang, Jinghong Zhao, Jianqin Niu, Chuan Chen, Junping Wang, Cheng Wang

**Affiliations:** ^1^ State Key Laboratory of Trauma and Chemical Poisoning, Institute of Combined Injury of PLA, College of Preventive Medicine Army Medical University Chongqing China; ^2^ Department of Nephrology, Xinqiao Hospital Army Medical University Chongqing China; ^3^ Department of Cancer Center, Daping Hospital Army Medical University Chongqing China; ^4^ Department of Gastroenterology, Chongqing Key Laboratory of Digestive Malignancies, Daping Hospital Army Medical University Chongqing China; ^5^ Biomedical Analysis Center Army Medical University Chongqing China; ^6^ Chongqing Key Laboratory of Neurobiology Chongqing China

**Keywords:** ferroptosis, gut microbiota, ionizing radiation‐induced intestinal injury, lipid metabolism, lithocholic acid, oligopeptide

## Abstract

Ionizing radiation‐induced intestinal injury (IRIII) reduces survival in nuclear accident victims and compromises the efficacy of abdominal radiotherapy, and current treatment options remain limited. Human defensin 5 (HD5)‐derived fragments are endogenous regulators of the gut microbiota, which affects host responses to radiation. However, whether these fragments influence intestinal radiosensitivity or can serve as lead compounds for IRIII therapeutics remains unclear. In this study, we investigated the role of HD5‐derived fragments in IRIII and developed AT9(C/G), a potent radioprotective oligopeptide based on the lead fragment AT9. Fecal metagenomic and metabolomic analyses revealed that the oral administration of AT9(C/G) enriches *Bifidobacterium pseudolongum* and increases lithocholic acid (LCA) levels in the intestine. Both murine and clinical studies demonstrated a negative correlation between IRIII severity and fecal LCA levels. The radioprotective effect of LCA was further validated in both mouse models and human small intestinal organoids. Mechanistically, LCA suppresses ferroptosis in irradiated cells by remodeling lipid metabolism. Specifically, LCA activates Takeda G protein‐coupled receptor 5 (TGR5), leading to the upregulation of sterol regulatory element‐binding protein 1 (SREBP1), which transcriptionally modulates stearoyl‐CoA desaturase 1 (SCD1) to catalyze monounsaturated fatty acid production. Pharmacological inhibition of SCD1 or genetic ablation of G‐protein coupled bile acid receptor 1 (*Gpbar1*, encodes TGR5) attenuates the protective effects of AT9(C/G) in mice. This study establishes that an oligopeptide can modulate gut microbiota‐derived LCA to confer intestinal radioprotection, presenting a promising preventive strategy against IRIII.

## Highlights

- Human defensin 5‐derived oligopeptide AT9(C/G) mitigates ionizing radiation‐induced intestinal injury.
- Oral administration of AT9(C/G) enriches *Bifidobacterium pseudolongum* and increases lithocholic acid (LCA) levels in the intestine.
- LCA activates Takeda G protein‐coupled receptor 5 (TGR5) and leads to the upregulation of sterol regulatory element‐binding protein 1 (SREBP1), which transcriptionally modulates stearoyl‐CoA desaturase 1 (SCD1) to catalyze monounsaturated fatty acid production, thereby suppressing ferroptosis in irradiated cells.

## Graphical abstract

AT9(C/G), an oligopeptide derived from human defensin 5, enriches *Bifidobacterium pseudolongum* and increases lithocholic acid (LCA) levels in the intestine. Reduced levels of LCA link to increased severity of ionizing radiation‐induced intestinal injury (IRIII) in both murine and clinical studies. Administration of LCA confers radioprotection across cells, mouse models, and human small intestinal organoids. Mechanistically, LCA activates Takeda G protein‐coupled receptor 5 (TGR5) and leads to the upregulation of sterol regulatory element‐binding protein 1 (SREBP1), which transcriptionally modulates stearoyl‐CoA desaturase 1 (SCD1) to catalyze monounsaturated fatty acid production, thereby suppressing ferroptosis in irradiated cells. Leveraging the radioprotective properties of LCA, AT9(C/G) effectively mitigates IRIII.

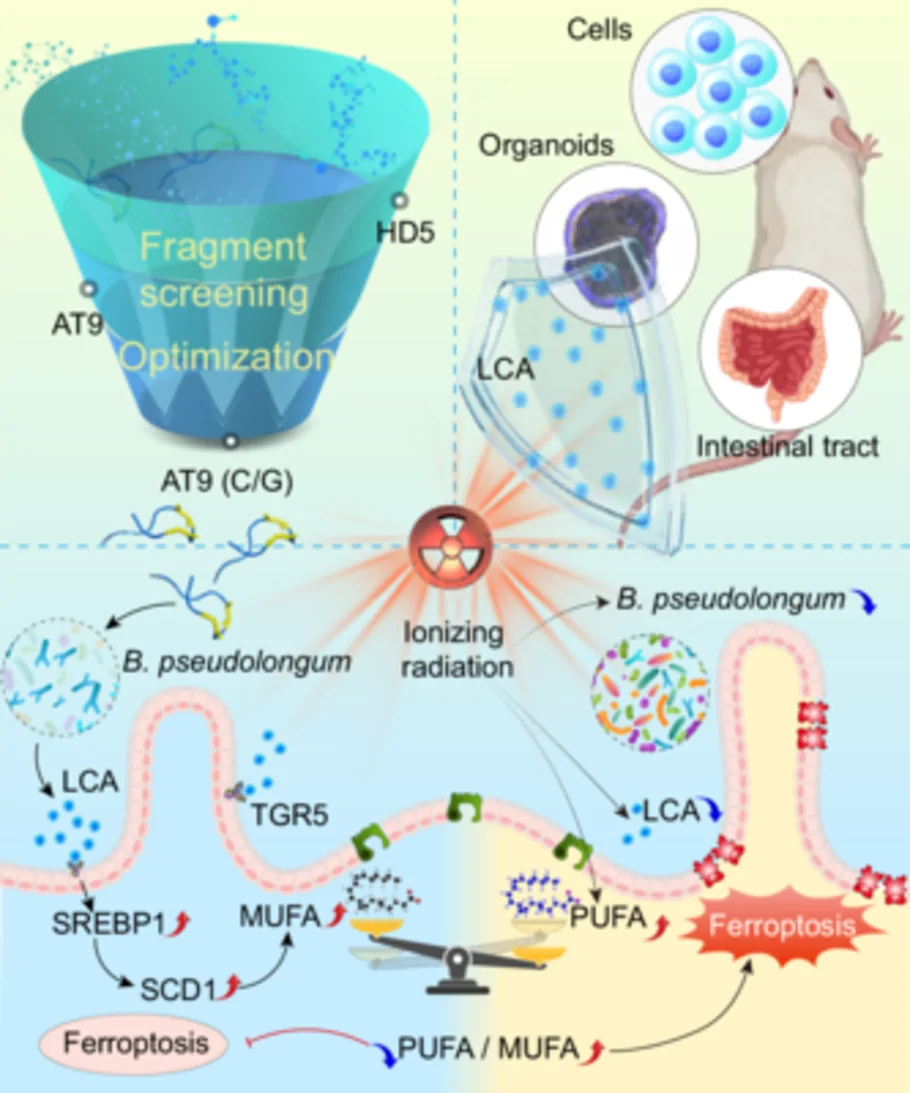

## INTRODUCTION

Nuclear energy and related technologies have been widely used in industrial, healthcare, and agricultural applications since the discovery of X‐rays by Roentgen in 1895. However, ionizing radiation (IR) is a double‐edged sword. The harmful effects of IR on the intestine and other organs were first documented as early as 1897 [1]. To date, the prevention and treatment of IR‐induced injuries remain unmet clinical needs. The mammalian intestine is among the most radiosensitive organs, and excessive exposure to IR can lead to lethal intestinal injury in humans. This condition primarily manifests as diarrhea, hematochezia, and severe endogenous infections [2], typically following exposure to high‐dose IR (>10 Gy). Currently, there are no documented cases of patients surviving accidental whole‐body IR at doses above 10 Gy. Additionally, IR‐induced intestinal injury (IRIII) occurs after radiotherapy for malignant tumors in the abdominal and pelvic cavities. Up to 90% of these patients develop acute gastrointestinal symptoms within 3 months after radiotherapy, which compromises both treatment efficacy and patient quality of life [3]. Unfortunately, no drugs have been approved by the FDA for IRIII treatment, highlighting the urgent need for developing effective countermeasures against this disease.

Investigating the mechanisms that underlie IRIII pathogenesis is critical for developing effective therapeutic strategies. Accumulating evidence indicates that, in addition to the inherent radiosensitivity of enterocytes, the gut microbiota significantly influences host radiation responses [4]. The human gut harbors approximately 100 trillion microorganisms, which form a complex ecosystem that is essential for maintaining intestinal homeostasis. Following abdominal irradiation, the microbial composition undergoes profound alterations, typically characterized by reduced diversity, depletion of beneficial probiotics (e.g., *Lactobacillus* and *Bifidobacterium*), and enrichment of pathogenic bacteria (e.g., *Escherichia coli* and *Staphylococcus*) [5]. These changes lead to decreased production of functional gut microbiota‐derived metabolites (GMMs), including bile acids (BAs) and short‐chain fatty acids [4, 6]. Consequently, gut microbiota‐targeted strategies, such as fecal microbiota transplantation (FMT) from healthy donors [7], probiotic supplementation [8], or the administration of microbiota regulators (e.g., enteric defensin peptides [2]), hold promise for mitigating IRIII.

As a member of the enteric defensin family, the α‐defensin human defensin 5 (HD5), produced by intestinal Paneth cells, is essential for maintaining the gut microbiota [9, 10]. Since our initial identification of linear HD5 in vivo [11], increasing evidence has shown that native HD5 undergoes proteolytic processing into shorter linear peptides, including AT28, TG26, CA23, and AT9, which actively modulate the microbial composition in the gut [12]. Compared with naturally structured HD5, these linear fragments are far more cost‐effective to synthesize, making them highly promising drug candidates. Characterizing these bioactive fragments can not only advance our understanding of mucosal immunity but also offer a strategic foundation for drug development. A prime example is D3, a synthetic 9‐amino‐acid peptide derived from AT9, which has been demonstrated to have therapeutic potential in alleviating obesity [13]. Although these HD5‐derived peptides are well established as endogenous modulators of the microbiota, their influence on intestinal radiosensitivity remains poorly understood. Moreover, their potential as lead compounds for developing IRIII therapeutics merits further exploration.

The pathological exacerbation of IRIII by dysbiosis of the microbiota involves the excessive activation of programmed cell death (PCD) pathways, including apoptosis, pyroptosis, and ferroptosis [14]. Ferroptosis is a widely studied type of PCD that is driven by excessive phospholipid (PL) peroxidation and serves as a critical mechanism through which IR energy is converted into cellular damage [15]. In intestinal epithelial cells, IR‐induced ferroptosis results from the dysregulation of multiple metabolic pathways involving iron transport [16], redox homeostasis [17], and lipid metabolism [3]. The latter critically regulates ferroptosis by controlling the balance between monounsaturated (MUFA‐PLs) and polyunsaturated fatty acyl‐containing phospholipids (PUFA‐PLs), which affects ferroptotic susceptibility [18]. Since MUFAs compete with PUFAs for incorporation into PLs and confer resistance to ferroptosis [19], targeting stearoyl‐CoA desaturase 1 (SCD1), the key enzyme that converts saturated fatty acids (SFAs) to MUFAs, has shown promise in mitigating IR‐induced skin injury [20]. Notably, SCD1 expression is modulated by GMMs, particularly by secondary BAs [21]. The biosynthesis of secondary BAs in the gut requires the sequential activity of distinct bacterial enzymes. This process begins with bile salt hydrolase (BSH)‐producing bacteria (e.g., *Lactobacillus* and *Bifidobacterium*) deconjugating primary BAs [22, 23], followed by 7α‐dehydroxylation of the resulting products by bacteria such as *Clostridium scindens* that express 7α‐dehydroxylase [24]. Consequently, modulating the gut microbiota to leverage the BA‐SCD1 axis against ferroptosis is a viable strategy for intestinal radioprotection.

In this study, we aimed to investigate the influence of HD5‐derived fragments on intestinal radiosensitivity and develop protective peptides based on the lead compound. By using a murine total abdominal irradiation (TAI) model, we identified the modified oligopeptide AT9(C/G), which has potent radioprotective properties. The mechanism by which AT9(C/G) ameliorates IRIII was probed using a multiomics approach integrating peptide–gut microbiota–host metabolic interactions. Our findings demonstrate that AT9(C/G) reshapes the gut microbiota and increases the intestinal levels of lithocholic acid (LCA). LCA is a secondary BA derived from microbial metabolism that has attracted attention for its role in metabolic regulation [25]. A key mechanism involves its activation on membrane‐bound Takeda G protein‐coupled receptor 5 (TGR5) [26], which can further modulate the expression of sterol regulatory element‐binding proteins (SREBPs) to reprogram cellular lipid metabolism [27]. We confirmed that AT9(C/G) exerts its radioprotective effects by activating the TGR5‐SREBP1‐SCD1 axis via LCA, thereby inhibiting IR‐triggered ferroptosis and ultimately mitigating IRIII. Furthermore, clinical investigations revealed a correlation between decreased LCA levels and IRIII severity in rectal cancer patients undergoing radiotherapy. These findings not only establish LCA as a potential predictive biomarker for early‐stage IRIII but also provide effective countermeasures for IRIII prevention.

## RESULTS

### The HD5‐derived oligopeptide AT9(C/G) efficiently alleviates IRIII

To investigate the effects of HD5 fragments on IR‐induced intestinal toxicity, we prepared four linear peptide fragments, AT28, TG26, CA23, and AT9 (Figure 1A), via solid‐phase chemical synthesis. Natural cyclic HD5 was employed as a control. Because males are more sensitive to IR than females [28], we used male mice for the initial peptide screening. The mice were orally administered these peptides (35 µM, 200 µL, once daily) for seven consecutive days before undergoing a single round of 12 Gy TAI with ^60^Co γ‐rays. The peptide dosage and TAI protocol were based on previous studies [2, 12]. Survival and intestinal symptoms were monitored for an additional 30 days after IR. While HD5 treatment increased mouse survival from 10% to 30% (*p* = 0.265, HR = 0.527, 95%CI: 0.171 to 1.63), AT28, TG26, and CA23 failed to confer protection (Figure 1B). Strikingly, the oligopeptide AT9 improved survival to 40% (*p* = 0.0893, HR = 0.371, 95%CI: 0.118 to 1.16). Further analysis revealed that, similar to HD5, AT9 alleviated IR‐induced symptoms, including diarrhea, hematochezia, and weight loss, by Day 4 post‐TAI. This led to a reduction in the mean disease activity index (DAI) score, although the difference compared to irradiated controls was not statistically significant (Figure 1C). Measurements of the length of the small intestine revealed that AT9 was comparable to HD5 in mitigating IR‐induced tissue shrinkage (Figure 1D). Histopathological examination of the ileum supported these findings, showing that AT9 reduced villous shedding (Figure 1E), preserved crypt counts, and attenuated villous shortening (Figure 1F).

**Figure 1:**
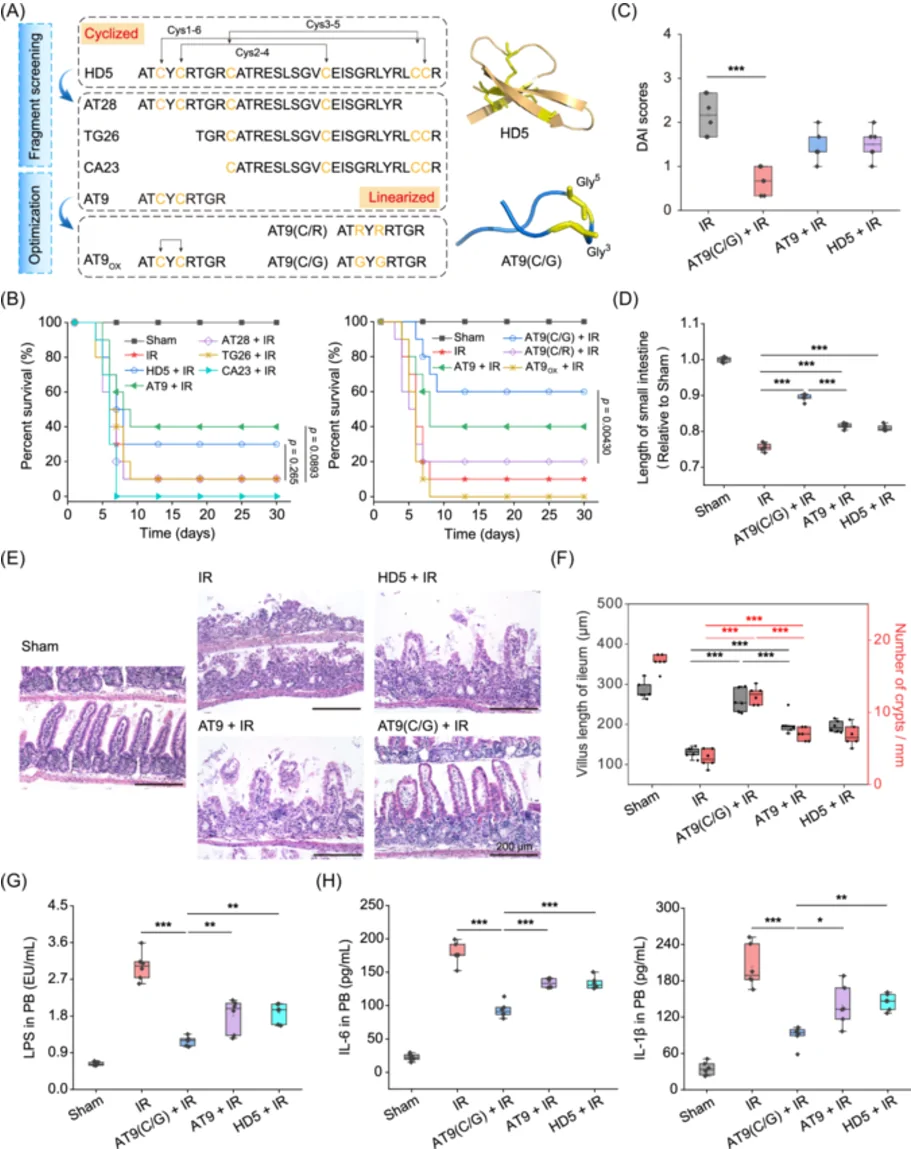
Human defensin 5 (HD5)‐derived oligopeptide AT9 and its derivative AT9(C/G) alleviate ionizing radiation‐induced intestinal injury (IRIII) in mice. (A) Amino acid sequences of the synthesized peptides. Cyclized HD5 (PDB: 1ZMP) and AT9(C/G) are presented as ribbon structures. The structure of AT9(C/G) was generated using PEPFOLD3. Cysteine residues are highlighted in yellow. (B) Survival rates of mice after total abdominal irradiation (TAI). Left panel: comparison of HD5 and its fragments of varying lengths (AT28, TG26, CA23, and AT9) administered post‐TAI. Right panel: comparison of AT9 and its derivatives (AT9(C/G), AT9(C/R), and AT9_OX_) administered post‐TAI. Mouse survival was monitored for 30 days post‐TAI. Each group contained 10 mice. (C) Disease activity index (DAI) scores of mice on day 4 after TAI. Results are presented as boxplots (*n* = 6 mice). ***, *p* < 0.001. (D) Relative length of the small intestine displayed as boxplots (*n* = 6 mice). ***, *p* < 0.001. (E) Hematoxylin and eosin (H&E) staining demonstrating the alleviative effects of AT9 and AT9(C/G) on IRIII in mice. Scale indicates 200 μm. (F) Villus length and number of crypts per millimeter in the ileum of mice. Results are shown as boxplots (*n* = 6 mice). ***, *p* < 0.001. (G) Lipopolysaccharide (LPS) levels in mouse peripheral blood (PB) on Day 3 post‐TAI. Results are shown as boxplots (*n* = 6 mice). **, *p* < 0.01; ***, *p* < 0.001. (H) Interleukin (IL)‐6 and IL‐1β contents in mouse PB on Day 3 post‐TAI. Results are displayed as boxplots (*n* = 6 mice). *, *p* < 0.05; **, *p* < 0.01; ***, *p* < 0.001.

The radioprotective efficacy and structural simplicity of AT9 make it an ideal lead candidate for treating IRIII. However, AT9 contains two cysteines (Cys^3^ and Cys^5^) that are susceptible to oxidation and can form an intramolecular disulfide bond, and the formation of this bond abolishes the ability of the peptide to improve survival in TAI‐exposed mice (Figure 1B). To increase the stability of AT9, we replaced cysteine (C) with glycine (G), an amino acid that can enhance protein structural stability [29], to generate the derivative AT9(C/G). Notably, this modified oligopeptide significantly increased mouse survival to 60% following lethal IR (Figure 1B; *p* = 0.00430, HR = 0.160, 95%CI: 0.0456 to 0.562). In contrast, another derivative, AT9(C/R), which was designed by replacing cysteine with arginine (R), a residue vulnerable to intestinal trypsin degradation [30], improved survival only marginally (20%). Additional analysis showed that oral administration of AT9(C/G) resulted in a significant reduction of the DAI score in mice with TAI (*p* < 0.001). Compared with AT9, AT9(C/G) demonstrated superior efficacy in preventing tissue shrinkage (Figure 1D) and preserving mucosal barrier integrity (Figure 1E,F). Furthermore, AT9(C/G) outperformed both AT9 and HD5 in reducing enterogenic LPS translocation (Figure 1G) and decreasing serum levels of proinflammatory cytokines (IL‐6 and IL‐1β; Figure 1H).

Notably, the superior efficacy of AT9 (C/G) over AT9 and HD5 in mitigating IRIII in female mice was also validated (Figure S1), which indicates a sex‐independent protective role for this oligopeptide. After verifying the radioprotective efficacy of AT9(C/G), we next assessed its biocompatibility. The results showed that a 1‐month course of AT9(C/G) had no significant impact on the body weight, blood cell counts, serous alanine aminotransferase, aspartate aminotransferase, creatinine, or urea levels in mice (Figure S2A–F). Additionally, histopathological analysis revealed no abnormalities in the major organs, including the intestine, heart, liver, spleen, lungs, and kidneys (Figure S2G). Collectively, these findings establish that the HD5‐derived fragment AT9 exhibits radioprotective properties and serves as a promising scaffold for drug development. The optimized derivative, AT9(C/G), emerges as a biocompatible and highly effective candidate for alleviating IRIII.

### AT9(C/G) alleviates IRIII by reshaping the gut microbiota

To elucidate the mechanism underlying AT9(C/G)‐mediated radioprotection, we conducted FMT experiments. Consistent with existing evidence that FMT ameliorates IRIII [31], our results demonstrated that transplantation of the microbiota from wild‐type mice increased the survival rate of irradiated mice to 30% (Figure 2A,B; *p* = 0.220, HR = 0.482, 95%CI: 0.150 to 1.55, relative to IR group). Notably, this protective effect was exceeded by FMT with microbiota from AT9(C/G)‐treated mice, which achieved a survival rate of 50% (*p* = 0.0663, HR = 0.323, 95%CI: 0.0964 to 1.08, relative to IR group). Further validation in pseudogerm‐free mice, which were established via oral administration of the broad‐spectrum antibiotic cocktail ABX, demonstrated complete abrogation of the protective effects of AT9(C/G) (Figure 2C). These findings underscore the essential role of remodeling of the gut microbiota in AT9(C/G)‐induced radioprotection.

**Figure 2:**
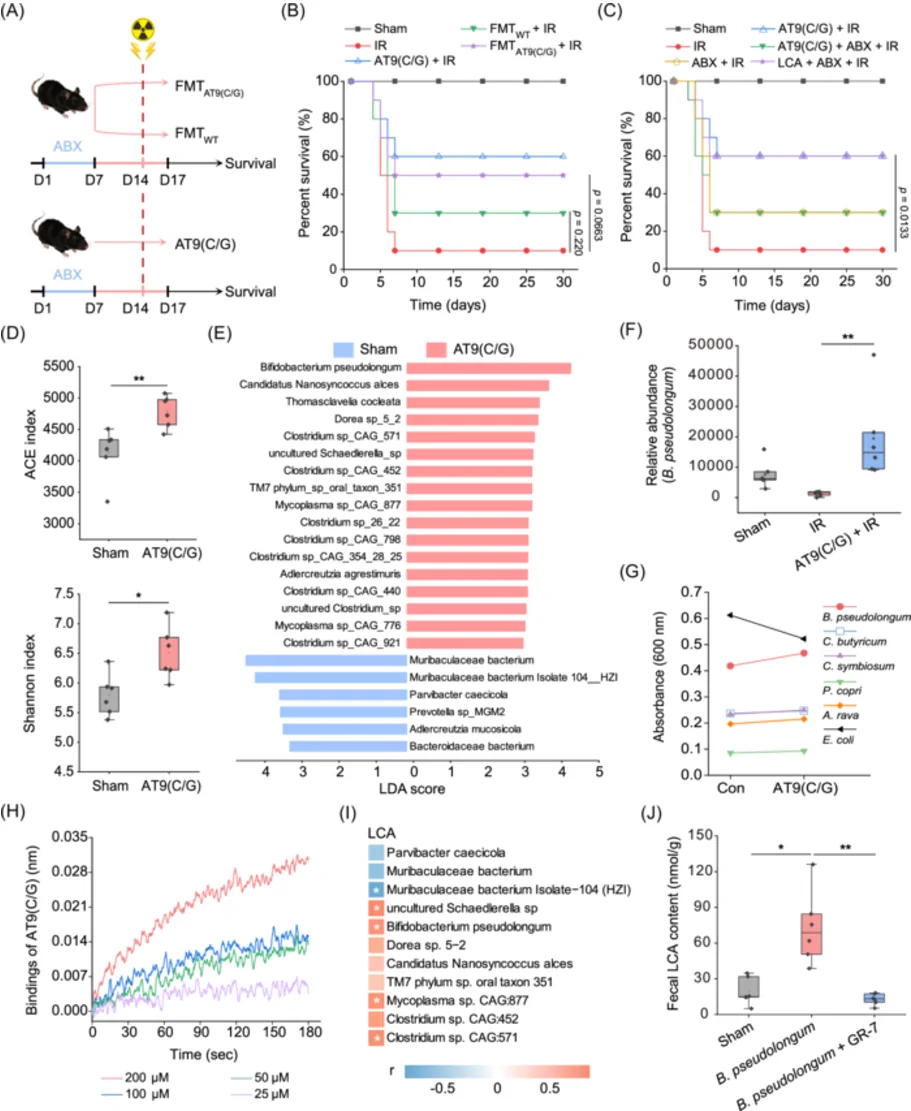
AT9(C/G) enriches *Bifidobacterium pseudolongum* (*B. pseudolongum*) and increases intestinal lithocholic acid (LCA) levels in mice. (A) Experimental workflow of antibiotic cocktail (ABX) administration and fecal microbiota transplantation (FMT). Mice received ABX from Days 1–7, followed by FMT from wild‐type (FMT_WT_) or AT9(C/G)‐treated donor mice (FMT_AT9(C/G)_) from Days 8 to 17, with survival monitored for 30 days post‐TAI. (B) Survival rates of FMT recipient mice following TAI. Mice received FMT from either wild‐type or AT9(C/G)‐treated donor mice, as shown in (A). Each group contained 10 mice. (C) Survival rates of ABX‐treated mice following TAI. ABX‐treated mice were administered LCA (15 mg/kg) for seven consecutive days to assess its effect on IRIII independently of the microbiota. Mouse survival was monitored for 30 days post‐TAI. Each group contained 10 mice. (D) α‐diversity indices (ACE and Shannon) of the gut microbiota in sham versus AT9(C/G)‐treated mice. Results are presented as boxplots (*n* = 6 mice). *, *p* < 0.05; **, *p* < 0.01. (E) Linear discriminant analysis effect size (LEfSe) analysis illustrating differentially abundant bacterial species between AT9(C/G)‐treated and sham groups. The logarithmic linear discriminant analysis (LDA) score threshold was set at 3. Species enriched in the AT9(C/G) group are shown in red, while those enriched in the sham group are shown in blue. (F) Relative abundance of *B. pseudolongum* in the gut microbiota of sham, IR, and AT9(C/G) + IR groups. Results are shown as boxplots (*n* = 6 mice). **, *p* < 0.01. (G) Absorbance of the bacterial suspension at 600 nm. The six bacterial strains were incubated for 16 h in the presence or absence of 35 μM AT9(C/G). Results represent the average of three independent experiments. (H) Biolayer interferometry (BLI) analysis showing real‐time binding kinetics of AT9(C/G) at various concentrations (25, 50, 100, and 200 μM) to immobilized biotinylated *B. pseudolongum* on streptavidin biosensors. (I) Heatmap showing the correlation between LCA concentration and the relative abundance of LEfSe‐enriched species in AT9(C/G)‐treated mice. *, *p* < 0.05, as determined by Spearman correlation analysis. The correlation coefficient threshold was set at 0.3. (J) Fecal LCA levels in mice administered *B. pseudolongum* with or without Gut Restricted‐7 (GR‐7) treatment. Results are shown as boxplots (*n* = 6 mice). *, *p* < 0.05; **, *p* < 0.01.

To investigate AT9(C/G)‐induced alterations in microbial composition, we performed metagenomic sequencing on fecal samples collected from mice 7 days after peptide administration. Significantly higher values of two α‐diversity indices, the ACE and Shannon indices, were observed for the samples from AT9(C/G)‐treated mice than for those from the sham group (Figure 2D). Nonparametric test analysis of similarities (ANOSIM) revealed substantial intergroup differences (*R* = 0.265, *p* = 0.015). Linear discriminant analysis effect size (LEfSe) analysis (LDA score > 3, *p* < 0.05) identified 17 bacterial taxa that were significantly enriched after AT9(C/G) treatment (Figure 2E). *Bifidobacterium pseudolongum* (*B. pseudolongum*), a common probiotic in the mammalian gut with potential roles in mitigating inflammatory bowel disease and age‐related cognitive decline [32, 33], showed the most pronounced enrichment (Table S1). The increased abundance of *B. pseudolongum* was maintained through day 3 post‐TAI (Figure 2F). In vitro assays using six bacterial strains preserved in our laboratory revealed that AT9(C/G) facilitated the growth of five anaerobic bacteria, namely, *B. pseudolongum*, *Clostridium symbiosum* (*C. symbiosum*), *Clostridium butyricum* (*C. butyricum*), *Prevotella copri* (*P. copri*), and *Alloprevotella rava* (*A. rava*) to varying degrees (Figure 2G), while inhibiting the growth of the opportunistic pathogen *Escherichia coli* (*E. coli*). Notably, AT9(C/G) exhibited a particularly marked growth‐promoting effect on *B. pseudolongum*. Biolayer interferometry (BLI) further confirmed that the peptide binds to immobilized *B. pseudolongum* in a dose‐dependent manner (Figure 2H). The direct uptake of fluorescein isothiocyanate (FITC)‐labeled AT9(C/G) by the probiotic was also visualized by confocal microscopy (Figure S3). Together, these results establish AT9(C/G) as a synthetic prebiotic with direct recruitment and growth‐promoting activity for *B. pseudolongum*.

### AT9(C/G) increases the radioprotective LCA in the gut

The gut microbiota generates a diverse repertoire of bioactive metabolites that critically regulate host physiology [23]. To investigate the effects of AT9(C/G) on GMMs, we conducted targeted metabolomic profiling using a Metabo‐Profile Q300 assay kit. Comparative analysis identified 13 differentially abundant metabolites in AT9(C/G)‐treated mice compared with the sham group (FC > 2 or < 0.5, VIP > 1, *p* < 0.05; Table S2), with three upregulated and ten downregulated metabolites (Figure 3A). On the basis of previous reports demonstrating the protective effects of elevated GMM levels against IRIII [34], we focused on the three metabolites with increased abundance: LCA, β‐deoxycholic acid (bDCA), and valerylcarnitine. Subsequent in vitro screening using irradiated (8 Gy γ‐ray) rat intestinal epithelial IEC‐6 cells revealed that LCA was the only radioprotectant among the metabolites (Figure 3B, Figure S4). The radioprotective efficacy of LCA was further validated in human intestinal epithelial HIEC‐6 cells (Figure 3B) and human small intestinal organoids (HSIOs; Figure 3C, Figure S5), where LCA treatment preserved the normal morphological characteristics and reduced the death of the organoids at 72 h post‐IR. Importantly, LCA did not confer radioprotection to the human colorectal carcinoma cell line HCT116 at the same drug concentrations (Figure S6), which points to its potential as a selective radioprotector.

**Figure 3:**
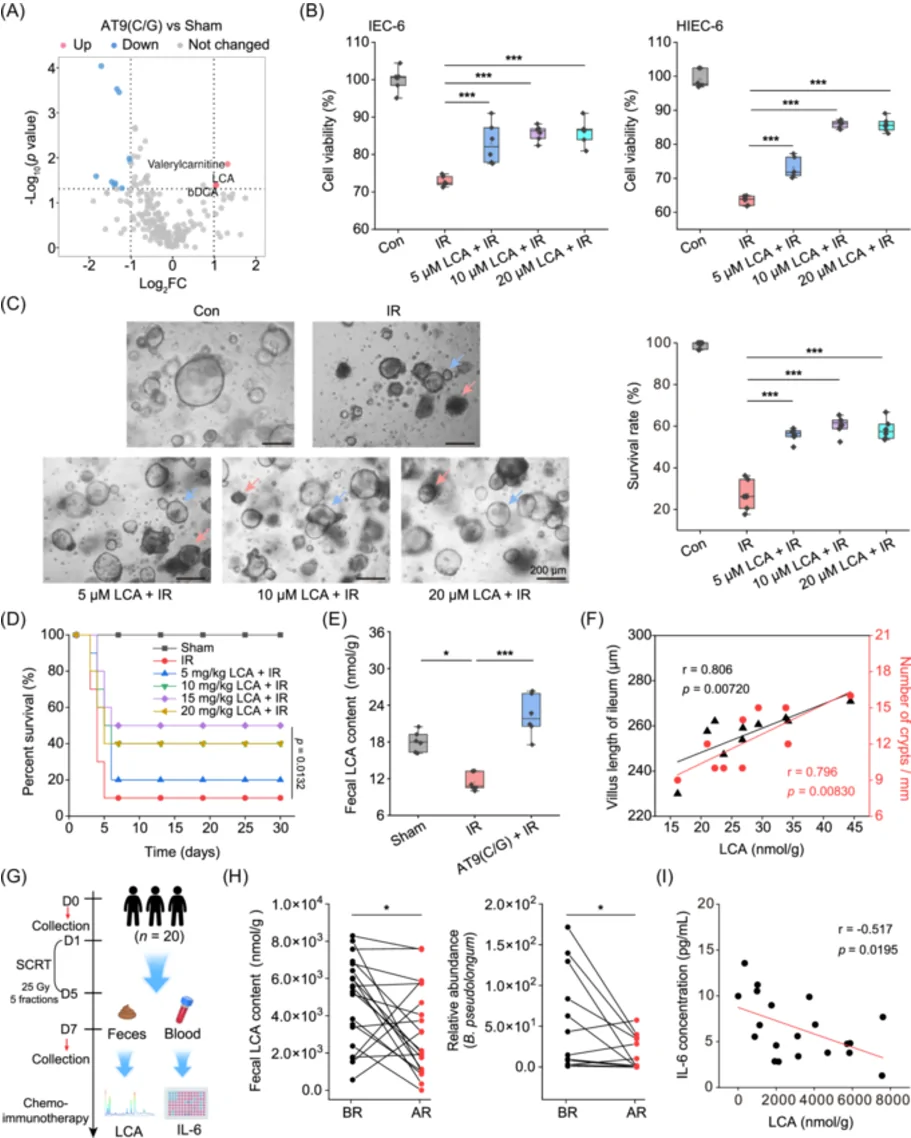
LCA reduces ionizing radiation (IR)‐induced intestinal toxicity in vitro and in vivo. (A) Volcano plot showing differentially abundant metabolites in the AT9(C/G) group compared to the Sham group, with red and blue representing upregulated and downregulated metabolites, respectively. All differential metabolites had variable importance in projection (VIP) scores > 1 in the orthogonal partial least squares‐discriminant analysis (OPLS‐DA) model. The model demonstrated strong explanatory and predictive power, as evidenced by cross‐validation results (*R*²*Y* = 0.936, *Q*²*Y* = 0.549) and further robust validation via permutation testing. (B) Viability of irradiated IEC‐6 and HIEC‐6 cells with or without LCA treatment. Results are presented as boxplots (*n* = 6 independent experiments). ***, *p* < 0.001. (C) Representative images of human small intestinal organoids (HSIOs) 72 h after IR exposure. Scale indicates 200 μm. Dead HSIOs were characterized by structural disintegration and the presence of dark fragmented debris. Representative dead and survival HSIOs are indicated by red and blue arrows, respectively. Survival rates of HSIOs are shown as boxplots (*n* = 6 donors). ***, *p* < 0.001. (D) Survival curves of mice with or without LCA treatment after TAI. Survival was monitored for 30 days post‐TAI. Each group contained 10 mice. (E) Fecal LCA contents in irradiated mice with or without AT9(C/G) treatment. Results are shown as boxplots (*n* = 6 mice). *, *p* < 0.05; ***, *p* < 0.001. (F) Correlation between fecal LCA content and ileal villus length or crypt number in AT9(C/G)‐treated mice after TAI. (G) Schematic of clinical study design. Rectal cancer patients (*n* = 20) received short‐course radiotherapy (SCRT; 25 Gy in 5 fractions over five consecutive days) followed by chemoimmunotherapy (CAPOX plus ivonescimab). Fecal samples were collected before SCRT (BR, Day 0) and on Day 7 after SCRT (AR) for LCA and *B. pseudolongum* analysis. Blood samples were collected at the same timepoints for serum IL‐6 measurement. (H) Fecal LCA levels and *B. pseudolongum* abundance in patients before and after short‐course radiotherapy (SCRT). Eight patients with undetectable *B. pseudolongum* at both time points were excluded from the analysis of its abundance variation. *, *p* < 0.05. (I) Correlation between fecal LCA content and serum IL‐6 levels in patients after SCRT.

To evaluate the efficacy of LCA for mitigating IRIII in vivo, we conducted TAI experiments in mice. Oral administration of LCA resulted in dose‐dependent radioprotective effects, with optimal survival rates (50%) achieved at a dosage of 15 mg/kg (Figure 3D; *p* = 0.0132, HR = 0.199, 95%CI: 0.0553 to 0.714). Pathological analysis corroborated the ability of LCA to mitigate ileal villous shedding, reduce crypt counts, and shorten villous length (Figure S7). Additionally, LCA supplementation increased the survival rate of ABX‐treated mice after TAI to 60% (Figure 2C; *p* = 0.0133, HR = 0.196, 95%CI: 0.0540 to 0.713), suggesting that LCA alleviates IRIII independently of the microbiota. Given the radioprotective effect of LCA, we next asked whether its abundance dictated the severity of IRIII. An examination of the fecal LCA content revealed significantly higher LCA concentrations in AT9(C/G)‐treated mice than in untreated mice after TAI (*p* < 0.001; Figure 3E). Consistent results were also obtained in mouse serum (Figure S8). Notably, association analysis demonstrated strong positive correlations between fecal LCA abundance and both villus length (*p* = 0.00720) and ileal crypt number (*p* = 0.00830; Figure 3F).

Since LCA is a secondary BA produced through gut microbiota‐mediated metabolism of primary BAs, we performed integrated metagenomic and metabolomic analyses to identify the source of LCA in AT9(C/G)‐treated mice. Four bacterial species, *B. pseudolongum*, *Clostridium CAG_571*, an uncultured *Schaedlerella* species, and *Mycoplasma CAG_877*, were significantly positively correlated with increased LCA contents (*p* < 0.05; Figure 2I). Given the predominance of *B. pseudolongum* in the gut microbiota of AT9(C/G)‐treated mice, we cultured *B. pseudolongum* and administered it to mice via oral gavage. After seven consecutive days of probiotic administration, the fecal LCA levels significantly increased (*p* = 0.0148; Figure 2J). However, co‐administration of Gut Restricted‐7 (GR‐7), a recognized BSH inhibitor [23], completely abolished this increase in LCA abundance. Collectively, these results demonstrate that gut microbiota‐derived LCA, which is enriched by AT9(C/G) treatment, mitigates IR‐induced intestinal epithelial damage in both in vitro and in vivo models.

To evaluate the clinical relevance of intestinal LCA levels in IRIII patients, we conducted a paired‐sample study of 20 rectal cancer patients receiving neoadjuvant short‐course radiotherapy (SCRT; 25 Gy/5 fractions) with chemoimmunotherapy at Daping Hospital, China (December 2024–June 2025; Figure 3G). Analysis of the fecal samples collected before and after SCRT revealed significant reductions in LCA levels after IR (*p* = 0.0237; Figure 3H), although the magnitude of decrease varied among individuals. Consistently, a marked reduction in *B. pseudolongum* abundance was determined in patients following SCRT (*p* = 0.0358). Furthermore, post‐SCRT fecal LCA levels showed a significant negative correlation with serum IL‐6 levels (*p* = 0.0195; Figure 3I), which increased with the severity of IRIII [35]. These clinical findings suggest the potential of LCA as a diagnostic biomarker for IRIII and provide evidence that the radioprotective mechanism of AT9(C/G) involves increasing the abundance of LCA.

### LCA reduces IR‐induced cell death by upregulating SCD1 expression to inhibit ferroptosis

To elucidate the mechanism underlying the radioprotective effects of LCA, we performed data‐independent acquisition proteomic analysis. Compared with cells treated with IR alone, IR‐exposed IEC‐6 cells treated with 10 µM LCA for 24 h presented differential expression of 364 proteins (FC > 1.5 or FC < 0.67, *p* < 0.05), with 220 upregulated and 144 downregulated. Kyoto Encyclopedia of Genes and Genomes (KEGG) pathway analysis of the differentially expressed proteins revealed significant enrichment of ferroptosis (Figure 4A; *p* = 0.00252). Ferroptosis is a form of PCD driven by iron accumulation and lipid peroxidation. To validate these ferroptotic features, we first performed transmission electron microscopy (TEM) and revealed that IR elicited mitochondrial morphology changes typical of ferroptosis, including a reduced number of mitochondrial cristae, mitochondrial shrinkage, and increased membrane density, whereas these morphological anomalies were ameliorated by LCA (Figure 4B). Consistently, C11‐BODIPY581/591 staining demonstrated reduced lipid peroxidation in irradiated cells after LCA treatment (Figure 4C, S9), a result further corroborated by decreased levels of lipid peroxidation byproducts, including 4‐hydroxynonenal (4‐HNE) and malondialdehyde (MDA; Figure 4D), both of which are known to promote ferroptosis [36]. As central hubs for cellular lipid and iron metabolism, lysosomes play a critical role in ferroptosis, where membrane lipid peroxidation induces permeabilization and subsequent iron leakage [37]. Our results confirmed that IR triggered marked lysosomal lipid peroxidation (Figure 4E) and the accumulation of intracellular labile ferrous ions (Fe²⁺; *p* < 0.001; Figure 4F). Notably, LCA treatment effectively mitigated all these aberrant changes. In HSIOs, LCA administration also alleviated the IR‐induced accumulation of Fe²⁺ and lipid peroxides (Figure 4G,H). Accordingly, LCA increased the viability of IEC‐6 cells and HSIOs in the presence of erastin (Figure 4I, Figure S10), a canonical ferroptosis inducer [38]. Taken together, these findings indicate that LCA suppresses IR‐induced ferroptosis.

**Figure 4:**
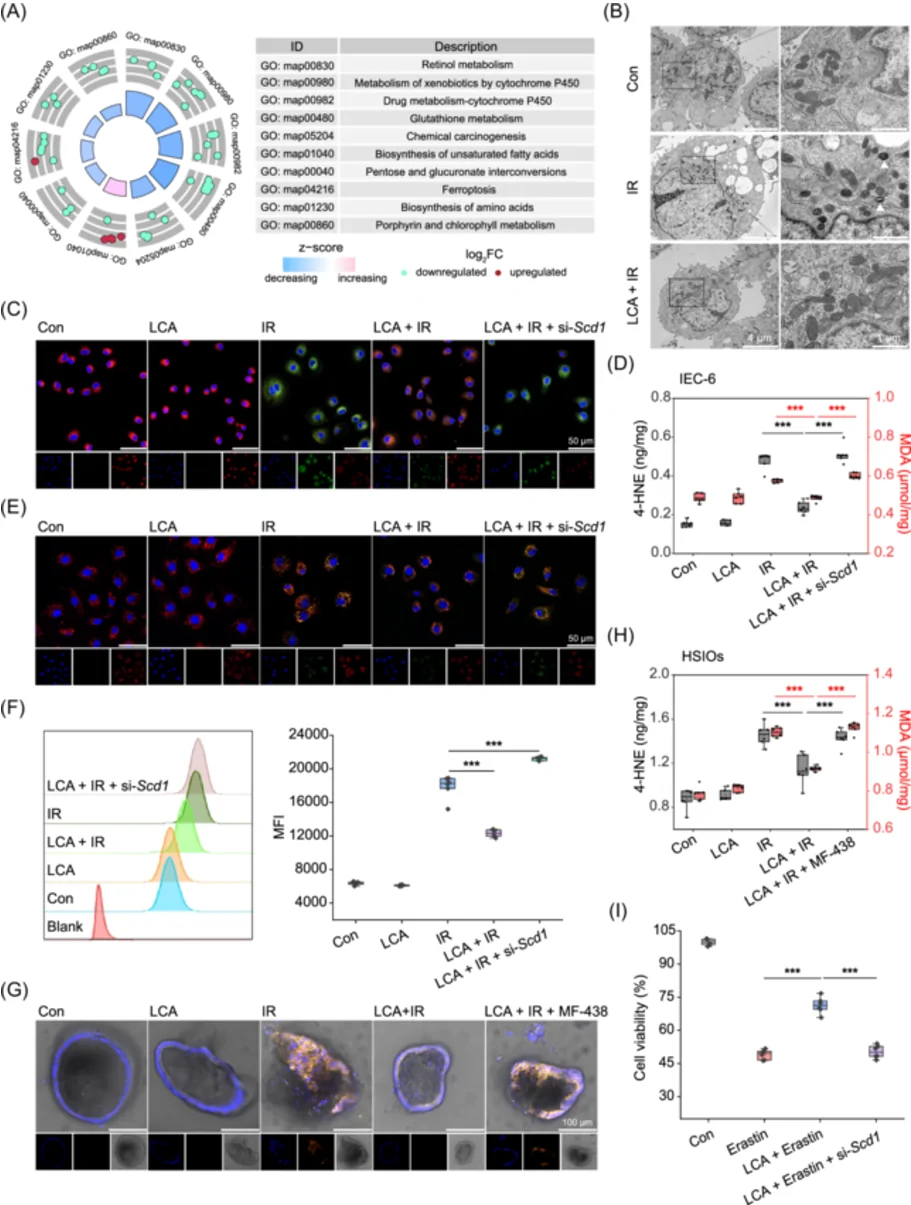
LCA inhibits IR‐induced ferroptosis by upregulating stearoyl‐CoA desaturase 1 (SCD1) expression. (A) Enrichment circle plot displaying the top 10 differential pathways modulated by LCA in irradiated IEC‐6 cells. Upregulated and downregulated proteins are shown in red and green, respectively. The inner circle ranges from blue to pink, representing *z*‐score values. (B) Transmission electron microscopy (TEM) images of IEC‐6 cells with or without IR or LCA treatment. Scale indicates 4 μm. Regions of interest are magnified in the right panel (scale bar, 1 μm). White arrows indicate representative mitochondrial changes. (C) C11‐BODIPY581/591 staining showing lipid peroxidation in IEC‐6 cells. Nuclei were stained with Hoechst 33342 (blue). Oxidized and non‐oxidized lipids exhibit green and red fluorescence, respectively. Colocalization appears as yellow. Scale indicates 50 μm. (D) 4‐hydroxynonenal (4‐HNE) and malondialdehyde (MDA) levels in cells. Results are presented as boxplots (*n* = 6 independent experiments). ***, *p* < 0.001. (E) Lipid peroxidation in lysosomes of IEC‐6 cells. Nuclei were stained with Hoechst 33342 (blue). Lysosomes were labeled with LysoTracker (red), and lipid peroxidation was detected using Liperfluo (green). Colocalization appears as yellow. Scale indicates 50 μm. (F) Flow cytometry analysis of intracellular Fe²⁺ levels in IEC‐6 cells. Results are displayed as boxplots (*n* = 6 independent experiments). ***, *p* < 0.001. (G) Confocal microscopy images showing Fe²⁺ accumulation (orange) in HSIOs. Nuclei were stained with Hoechst 33342 (blue). Scale bar indicates 100 μm. (H) Quantification of 4‐HNE and MDA levels in HSIOs treated with SCD1 inhibitor MF‐438. Results are shown as boxplots (*n* = 6 donors). ***, *p* < 0.001. (I) Viability of IEC‐6 cells treated with 5 µM erastin and/or 10 µM LCA, with or without *Scd1* knockdown. Results are presented as boxplots (*n* = 6 independent experiments). ***, *p* < 0.001.

Ferroptosis depends on the peroxidation of membrane PLs, and the levels of PLs containing PUFAs, MUFAs, or SFAs dictate ferroptosis sensitivity [39]. Given that KEGG analysis also revealed significant enrichment of unsaturated fatty acid (UFA) biosynthesis signaling (Figure 4A), we performed targeted lipidomics analysis of IEC‐6 cells to investigate whether altered UFA production contributes to LCA‐mediated inhibition of ferroptosis. We first analyzed 17 free fatty acids (Figure 5A) and reported that IR significantly increased the intracellular levels of arachidonic acid (AA, 20:4; *p* < 0.001) and adrenic acid (AdA, 22:4; *p* < 0.001; Figure 5B), two key PUFAs that promote ferroptosis [40]. However, treatment with 10 µM LCA for 24 h suppressed the IR‐induced upregulation of AA and AdA while markedly increasing the levels of palmitoleic acid (POA, 16:1; *p* < 0.001) and oleic acid (OA, 18:1; *p* < 0.001), two MUFAs known to inhibit ferroptosis [19]. Consistent with these results, compared with sham‐treated cells, irradiated cells exhibited higher levels of phosphatidylethanolamines (PEs) containing AA or AdA at the sn‐2 position (e.g., PE 16:0_20:4 and PE 18:0_22:4) (Figure 5C). LCA reversed these changes, significantly increasing the levels of PE‐MUFA species such as PE 16:1_16:1 (*p* = 0.0138) and PE 18:1_18:1 (*p* < 0.001). Similar trends were observed for six other membrane lipid classes, including phosphatidylinositols (PIs), phosphatidylglycerols (PGs), phosphatidylcholines (PCs), phosphatidylserines (PSs), phosphatidic acids (PAs), and bis(monoacylglycerol)phosphates (BMPs; Figure S11). Notably, IR increased the PUFA:MUFA ratio in these PLs, whereas LCA restored the balance between MUFAs and PUFAs (Figure 5D). These findings suggest that LCA reprograms lipid metabolism toward MUFA‐enriched PLs and away from proferroptotic PUFA‐PLs.

**Figure 5:**
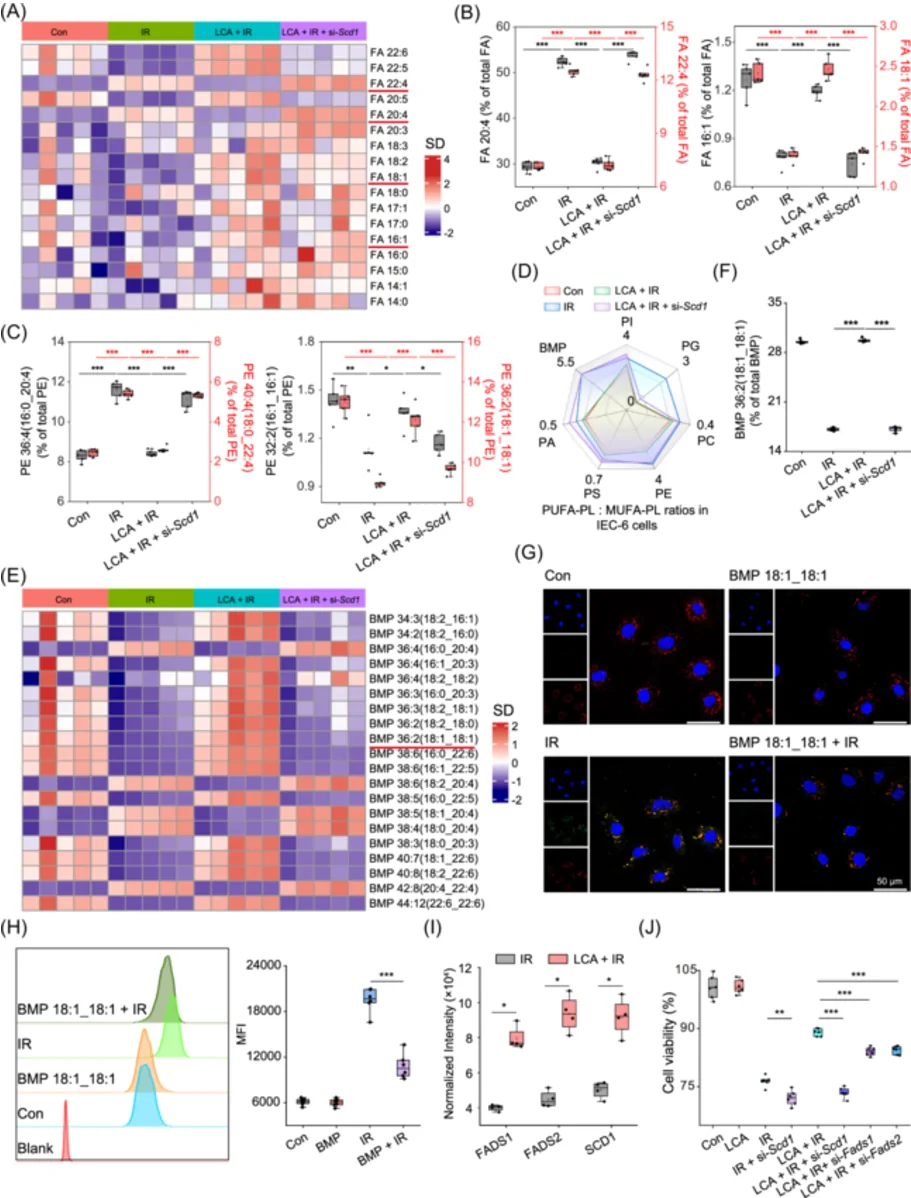
LCA reprograms lipid metabolism in irradiated cells by upregulating SCD1 expression. (A) Heatmap showing the effects of IR and LCA on free fatty acid (FA) levels in IEC‐6 cells. Each group contains five independent samples. Arachidonic acid (AA), adrenic acid (AdA), palmitoleic acid (POA), and oleic acid (OA) are underlined. (B) Relative abundances of AA, AdA, POA, and OA in IEC‐6 cells. Results are shown as boxplots. ***, *p* < 0.001. (C) Relative abundances of phosphatidylethanolamine (PE) 36:4, PE 40:4, PE 32:2, and PE 36:2 in IEC‐6 cells. Results are shown as boxplots. *, *p* < 0.05; **, *p* < 0.01; ***, *p* < 0.001. (D) Radar plots illustrating the proportion of polyunsaturated fatty acid (PUFA) versus monounsaturated fatty acid (MUFA) in PE, phosphatidylcholine (PC), phosphatidylglycerol (PG), phosphatidylinositol (PI), phosphatidic acid (PA), phosphatidylserine (PS), and bis(monoacylglycerol)phosphate (BMP). The data represent mean values from five samples. (E) Heatmap depicting the effects of IR and LCA on levels of BMP‐phospholipids (PLs) containing FAs in IEC‐6 cells. BMP 18:1_18:1 is underlined. (F) Relative abundance of BMP 18:1_18:1 in IEC‐6 cells. Results are shown as boxplots. ***, *p* < 0.001. (G) Effect of BMP 18:1_18:1 administration on lysosomal lipid peroxidation in IEC‐6 cells. Nuclei were stained with Hoechst 33342 (blue). Lysosomes were labeled with Lyso‐Tracker (red), and lipid peroxidation was detected using Liperfluo (green). Colocalization appears as yellow. Scale indicates 50 μm. (H) Flow cytometry analysis of intracellular Fe²⁺ levels. Results are presented as boxplots (*n* = 6 independent experiments). ***, *p* < 0.001. (I) Normalized intensities of fatty acid desaturase 1 (FADS1), fatty acid desaturase 2 (FADS2), and SCD1 in irradiated IEC‐6 cells with or without LCA treatment. Results are shown as boxplots (*n* = 4 independent samples). *, *p* < 0.05. (J) Viability of IEC‐6 cells following IR and/or LCA treatment under *Scd1*, *Fads1*, or *Fads2* modulation. Results are shown as boxplots (*n* = 6 independent experiments). **, *p* < 0.01; ***, *p* < 0.001.

Among the lipids remodeled by LCA in irradiated cells, BMPs undergo particularly noteworthy alterations. As a major PL component of the intraluminal membranes within late endosomes and lysosomes, BMP contributes to lysosomal membrane integrity and function [41]. Given that LCA treatment reduced the PUFA:MUFA ratio in BMPs, a change that mirrored its mitigation of IR‐induced lysosomal lipid peroxidation, we hypothesized that the observed increase in BMP‐MUFA species, such as BMP 18:1_18:1 (Figure 5E,F), might be responsible. To test this, we supplemented cells with 50 µM BMP 18:1_18:1 and found that this BMP‐MUFA not only attenuated lysosomal lipid peroxidation (Figure 5G) but also significantly suppressed the IR‐induced elevation of labile Fe²⁺ levels (*p* < 0.001; Figure 5H). Therefore, BMP‐MUFA likely acts as a downstream effector of LCA, reducing lysosomal iron leakage and thereby contributing to the inhibition of IR‐induced ferroptosis.

Consistent with the observed changes in intracellular UFAs following LCA treatment, InterPro enrichment analysis of the proteomic data revealed the role of fatty acid desaturases in this process (Figure S12). Specifically, LCA upregulated the expression of three fatty acid desaturases in irradiated cells: SCD1, fatty acid desaturase 1 (FADS1), and fatty acid desaturase 2 (FADS2; Figure 5I). These findings were further confirmed by reverse transcription quantitative PCR (RT–qPCR; Figure S13). We also examined the transcription levels of three ferroptosis‐related genes, including glutathione peroxidase 4 (*Gpx4*), nuclear factor erythroid‐2‐related factor 2 (*Nfe2l2*), and acyl‐coenzyme A (CoA) synthetase long‐chain family member 4 (*Acsl4*). While LCA altered the expression of *Acsl4*, it had no effect on the transcription of *Gpx4* or *Nfe2l2*. Nevertheless, the change in *Acsl4* expression was not as prominent as the transcriptional upregulation observed for the three fatty acid desaturases (Figure S13). To investigate the functional roles of SCD1, FADS1, and FADS2 in LCA‐mediated radioprotection, we individually silenced these proteins in IEC‐6 cells using small interfering RNA (siRNA; Figure S14). The results showed that the upregulation of each contributes to the ability of LCA to reduce IR‐induced cell death, with SCD1 exhibiting the most pronounced effect (Figure 5J). Supporting this observation, *Scd1* knockdown increased IEC‐6 cell radiosensitivity independent of LCA treatment. Given that SCD1 catalyzes MUFA synthesis from SFAs [20], we hypothesized that LCA‐induced SCD1 upregulation might drive the observed increase in MUFA‐PLs. Lipidomics analysis confirmed that *Scd1* silencing attenuated the ability of LCA to increase MUFA and MUFA‐PL levels and restored the high PUFA:MUFA ratio in PLs after IR (Figure 5A–F, Figure S11). Consequently, *Scd1* knockdown abolished the inhibitory effects of LCA on both lipid peroxidation and Fe^2+^ accumulation in irradiated cells (Figure 4C–F). Consistent results were obtained upon the administration of the SCD1 inhibitor MF‐438 in HSIOs (Figure 4G,H). LCA thus no longer protected against erastin‐induced cell death upon *Scd1* knockdown (Figure 4I). Notably, exogenous OA supplementation mitigated the adverse effects of *Scd1* silencing on the anti‐ferroptotic and cytoprotective activities of LCA (Figure S15). These results collectively establish that SCD1 upregulation is essential for the ability of LCA to inhibit IR‐induced ferroptosis and prevent cell death.

### LCA upregulates SCD1 expression by activating TGR5‐SREBP1 signaling in vitro and in vivo

LCA serves as an endogenous ligand for both the membrane‐bound G protein‐coupled receptor TGR5 and several nuclear receptors, including the farnesoid X receptor (FXR), vitamin D receptor (VDR), and constitutive androstane receptor (CAR) [42]. To investigate the roles of these receptors in LCA‐mediated radioprotection, we performed receptor‐specific silencing in IEC‐6 cells using siRNA (Figure S14). While genetic interference with nuclear receptor subfamily 1 group H member 4 (*Nr1h4*, encodes FXR), *Vdr*, or nuclear receptor subfamily 1 group I member 3 (*Nr1i3*, encodes CAR) had no significant effect on the mitigating effect of LCA, G‐protein coupled bile acid receptor 1 (*Gpbar1*, encodes TGR5) knockdown abolished the ability of LCA to attenuate IR‐induced cell death (Figure 6A). Similarly, pharmacological inhibition of TGR5 using the antagonist SBI‐115 abrogated LCA‐mediated protection against IR (Figure 6B). Most importantly, *Gpbar1* deficiency significantly prevented LCA‐induced SCD1 upregulation, as demonstrated by both RT‐qPCR (*p* < 0.001; Figure 6C) and immunofluorescence analysis (Figure S16), indicating that LCA regulates SCD1 expression through a TGR5‐dependent mechanism.

**Figure 6:**
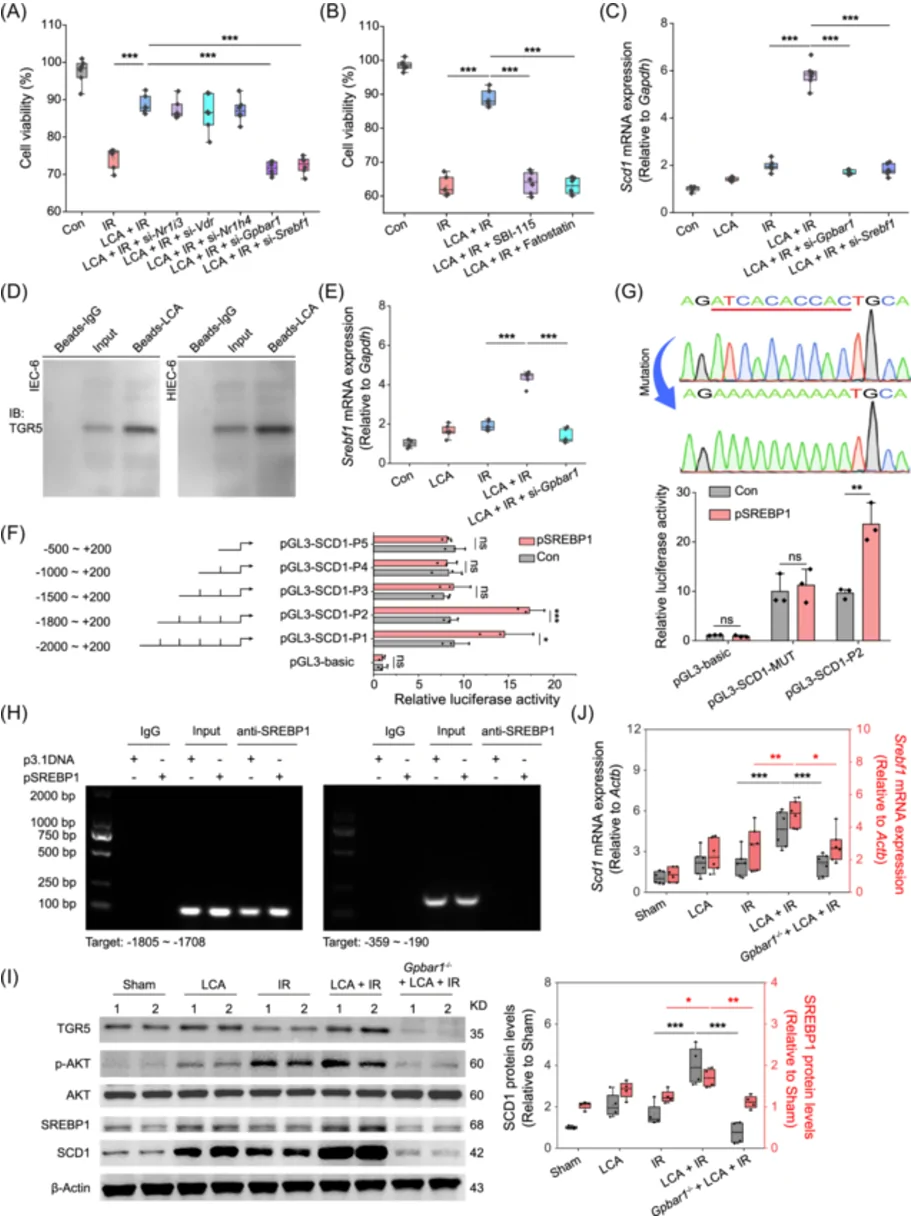
LCA upregulates SCD1 expression via the Takeda G protein‐coupled receptor 5 (TGR5)–sterol regulatory element‐binding protein 1 (SREBP1) signaling axis. (A) Viability of IEC‐6 cells after IR and/or LCA treatment under modulation of nuclear receptor subfamily 1 group H member 4 (*Nr1h4*), vitamin D receptor (*Vdr*), nuclear receptor subfamily 1 group I member 3 (*Nr1i3*), G‐protein coupled bile acid receptor 1 (*Gpbar1*), or sterol regulatory element binding transcription factor 1 (*Srebf1*). Results are shown as boxplots (*n* = 6 independent experiments). ***, *p* < 0.001. (B) Viability of IEC‐6 cells treated with IR and/or LCA in the presence of SBI‐115 or fatostatin. Results are displayed as boxplots (*n* = 6 independent experiments). ***, *p* < 0.001. (C) *Scd1* mRNA expression in IEC‐6 cells after IR and/or LCA treatment under modulation of *Gpbar1* or *Srebf1*. Results are shown as boxplots (*n* = 6 independent experiments). ***, *p* < 0.001. (D) Detection of LCA interaction with TGR5 by streptavidin‐bead pull‐down assay. Lysates from cells treated with biotinylated‐LCA or biotinylated‐IgG were incubated with streptavidin magnetic beads to collect target proteins. The precipitates were subsequently analyzed by immunoblotting with an anti‐TGR5 antibody. (E) *Srebf1* mRNA expressions in IEC‐6 cells following IR and/or LCA treatment in the absence or presence of *Gpbar1* knockdown. Results are shown as boxplots (*n* = 6 independent experiments). ***, *p* < 0.001. (F) Results of dual‐luciferase reporter assays. Five truncated *SCD1* promoter constructs (P1–P5) were transfected into HIEC‐6 cells, and their activity was assessed by dual‐luciferase assay following 24 h of control or SREBP1 overexpression treatment. ns, not significantly; *, *p* < 0.05; ***, *p* < 0.001. (G) Relative luciferase activity in HIEC‐6 cells transfected with pGL3‐SCD1‐P2 or pGL3‐SCD1‐MUT. ns, not significantly; **, *p* < 0.01. (H) Chromatin immunoprecipitation (ChIP) analysis of SREBP1 binding to the *Scd1* promoter. Precipitated DNA was amplified by polymerase chain reaction (PCR) using primers flanking the putative SREBP1‐binding site (−1805 to −1708) and a negative‐control region (−359 to −190) in the *Scd1* promoter. Input DNA (1%) served as the positive control. A representative agarose gel image is shown. (I) Protein levels of TGR5, p‐protein kinase B (AKT), AKT, SREBP1, and SCD1 in mouse ileum after LCA treatment. β‐Actin was used as the reference. Relative abundances of SCD1 and SREBP1 based on densitometric analysis are shown as boxplots (*n* = 4 mice). *, *p* < 0.05; **, *p* < 0.01; ***, *p* < 0.001. (J) *Scd1* and *Srebf1* mRNA expression levels in the ileum of wild‐type and *Gpbar1*‐knockout mice following IR and/or LCA treatment. Results are shown as boxplots (*n* = 6 mice). *, *p* < 0.05; **, *p* < 0.01; ***, *p* < 0.001.

To explore how TGR5 mediates the regulation of SCD1 by LCA, we first performed a streptavidin‐bead pull‐down assay followed by immunoblotting. The results confirmed the recruitment of LCA to TGR5 in both IEC‐6 and HIEC‐6 cells (Figure 6D), which allows LCA‐induced activation of the TGR5 signaling pathway. Since TGR5 activation modulates SREBP levels via the protein kinase B (AKT) signaling pathway [43], and because SCD1 expression is regulated by SREBP1 [44], we next hypothesized that SREBP1 might mediate the upregulation of SCD1 by LCA following TGR5 activation. Subsequent experiments in irradiated IEC‐6 cells confirmed that LCA increased SREBP1 expression at both the transcriptional (Figure 6E) and protein levels (Figure S16). In contrast, genetic interference with sterol regulatory element binding transcription factor 1 (*Srebf1*, encodes SREBP1) impaired the ability of LCA to promote *Scd1* expression (Figure 6C). Accordingly, either *Srebf1* knockdown or treatment with 10 μM fatostatin, a small‐molecule inhibitor of SREBPs [45], diminished the radioprotective effect of LCA (Figure 6A,B). Moreover, in line with the suppression of SCD1 expression upon TGR5 silencing, *Gpbar1* knockdown attenuated LCA‐induced *Srebf1* upregulation in irradiated IEC‐6 cells (Figure 6E).

Leveraging the advantage of the well‐annotated human genome, we employed an integrated approach combining luciferase reporter and chromatin immunoprecipitation (ChIP) assays in HIEC‐6 cells to determine whether SREBP1 binds directly to the *SCD1* promoter. Five truncated SCD1 reporter plasmids and a SREBP1 overexpression plasmid were constructed (Figure S17) and transfected into HIEC‐6 cells. As shown in Figure 6F, SREBP1 overexpression markedly increased the luciferase activities of the reporter plasmids pGL3‐SCD1‐P1 (−2000 to +200) and pGL3‐SCD1‐P2 (−1800 to +200), but not pGL3‐SCD1‐P3 (−1500 to +200), pGL3‐SCD1‐P4 (−1000 to +200) or pGL3‐SCD1‐P5 (−500 to +200), indicating that an SREBP1 response element is located within the region from −2000 to −1500 nucleotides relative to the transcriptional start site. Mutation of the top predicted SREBP1 binding site from JASPAR (−1737 to −1728; Table S3) within this region abolished the activation of the *SCD1* promoter (Figure 6G). Furthermore, the ChIP assay validated the direct binding of SREBP1 to the *SCD1* promoter region (−1805 to −1708; Figure 6H), whereas no enrichment was observed at a proximal region (−359 to −190). These results indicate that SREBP1 can promote the expression of *SCD1* by binding to its promoter region.

To determine whether LCA induces SREBP1 and SCD1 upregulation in vivo and whether this effect depends on TGR5, we next performed TAI experiments using *Gpbar1* gene knockout (*Gpbar1* ^−/−^) mice. Immunoblotting revealed that oral administration of LCA (15 mg/kg) increased the expression of SCD1 and its upstream regulators, SREBP1 and phosphorylated AKT (p‐AKT), in ileum tissues, particularly in TAI‐exposed mice (Figure 6I, Figure S18A). However, in *Gpbar1* ^−/−^ mice, LCA failed to modulate p‐AKT, SREBP1, or SCD1 expression. Consistent with these protein‐level observations, *Gpbar1* knockout also suppressed the transcriptional upregulation of *Scd1* and *Srebf1* induced by LCA (Figure 6J). These findings collectively demonstrate that LCA upregulates SCD1 expression by activating the TGR5‐SREBP1 signaling axis.

### SCD1 inhibition or *Gpbar1* knockout diminishes the radioprotective effect of AT9(C/G) in mice

Given that LCA is enriched in the gut of AT9(C/G)‐treated mice, we speculated that the modulatory effect of LCA on lipid metabolism and ferroptosis through TGR5‐mediated upregulation of SCD1 might be involved in the intestinal radioprotection of AT9(C/G). To test this hypothesis, we first performed transcriptome sequencing to analyze the effects of AT9(C/G) administration on biological processes in the ileal tissues of irradiated mice. Gene ontology‐biological process (GO‐BP) annotations of the differentially expressed genes (FC > 1.5 or FC < 0.67, *p* < 0.05; 2278 upregulated and 2831 downregulated) revealed significant enrichment of lipid metabolic process (Figure 7A), among which *Scd1* was among the most markedly upregulated genes (Figure 7B). These sequencing data were further validated by RT‐qPCR (Figure 7C) and immunoblotting analyses (Figure 7D,E), both of which revealed that AT9(C/G) administration increased SCD1 expression in ileal tissues, particularly in TAI‐exposed mice. Consistent with the role of SCD1 upregulation in shifting lipid metabolism toward MUFAs and away from PUFAs, treatment with AT9(C/G) significantly decreased the content of proferroptotic AA (20:4; *p* = 0.00140) but increased the OA (18:1; *p* < 0.001) level in irradiated mice (Figure 7F), thereby reducing the levels of the lipid peroxidation byproducts, 4‐HNE (*p* < 0.001; Figure 7G) and MDA (*p* < 0.001; Figure 7H), as well as Fe accumulation (*p* < 0.001; Figure 7I). Strikingly, oral administration of MF‐438 abrogated the ability of AT9(C/G) to mitigate the accumulation of AA, lipid peroxides, and Fe in the ileum of mice subjected to TAI (Figure 7F–I). Consequently, pharmacological inhibition of SCD1 attenuated the protective effect of AT9(C/G) against IR‐induced mucosal barrier disruption (Figure 7J, Figure S19).

**Figure 7:**
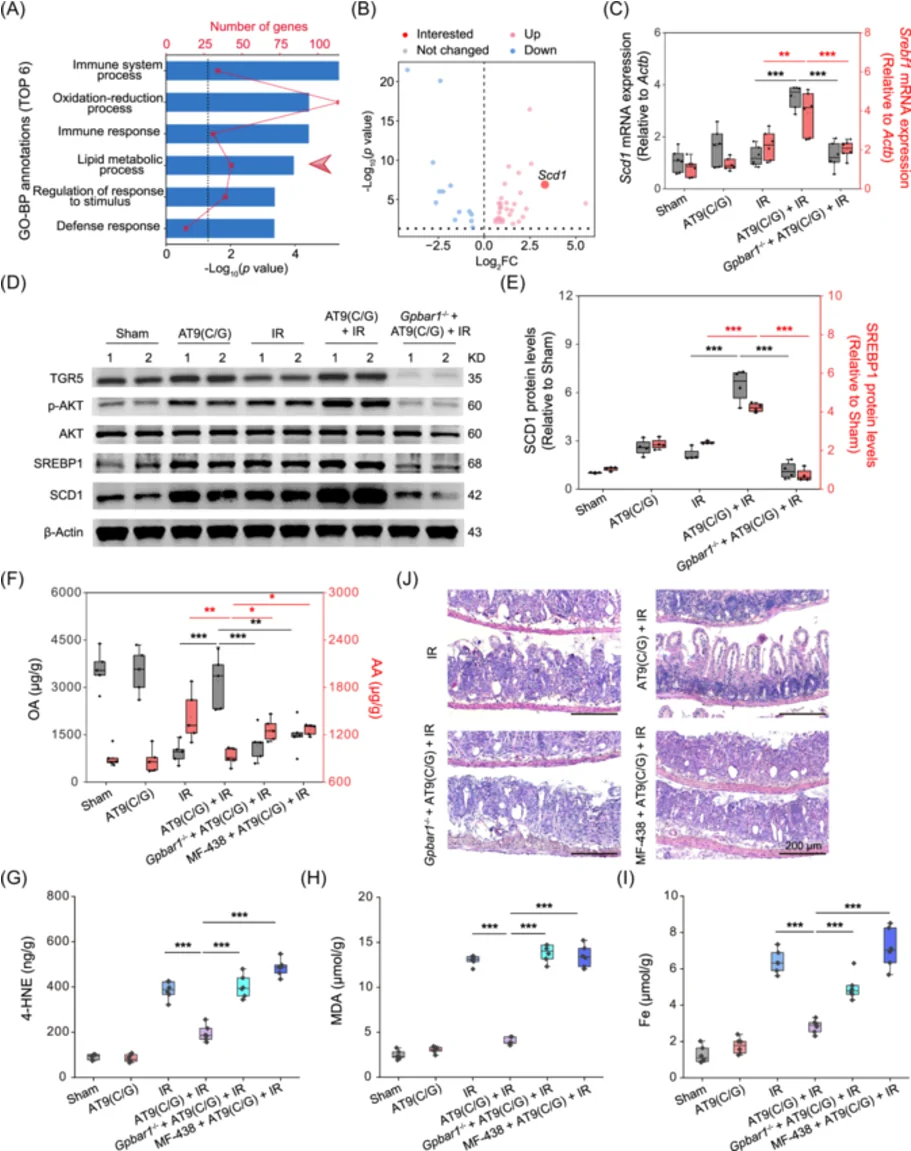
Activation of the TGR5‐SREBP1‐SCD1 axis mediates the radioprotective effect of AT9(C/G) in intestine. (A) The top six significantly enriched gene ontology‐biological process (GO‐BP) annotations in ileal tissues, with lipid metabolic process highlighted. (B) A volcano plot of differentially expressed genes related to lipid metabolic process in ileal tissues from AT9(C/G)‐treated and irradiated mice. (C) Effects of TAI and/or AT9(C/G) on ileal *Scd1* and *Srebf1* expression. Results are shown as boxplots (*n* = 6 mice). **, *p* < 0.01; ***, *p* < 0.001. (D) Protein bands of TGR5, p‐AKT, AKT, SREBP1, and SCD1 in mouse ileum after AT9(C/G) treatment. β‐Actin serves as the reference. (E) Quantification of protein levels of SCD1 and SREBP1 in mouse ileum after AT9(C/G) treatment. Results are shown as boxplots (*n* = 4 mice). ***, *p* < 0.001. (F) Arachidonic acid (AA) and oleic acid (OA) levels in ileal tissues of mice. Results are presented as boxplots (*n* = 5 mice). *, *p* < 0.05; **, *p* < 0.01; ***, *p* < 0.001. (G‐I) 4‐HNE (G), MDA (H), and total Fe (I) levels in ileal tissues of mice. Results are displayed as boxplots (*n* = 6 mice). ***, *p* < 0.001. (J) H&E staining showing that SCD1 inhibition or *Gpbar1* knockout diminishes the protective effect of AT9(C/G) against IRIII in mice. Scale indicates 200 μm.

To investigate whether TGR5 mediated SCD1 upregulation after AT9(C/G) treatment, we next performed a TAI experiment using *Gpbar1* ^−/−^ mice. Consistent with the influence of *Gpbar1* silencing in irradiated cells, *Gpbar1* knockout significantly suppressed the ability of AT9(C/G) to upregulate the expression of SCD1 and its upstream effectors SREBP1 and p‐AKT in TAI‐exposed mice (Figure 7D,E, Figure S18B). The inhibitory effect of *Gpbar1* knockout on *Scd1* and *Srebf1* expression was also confirmed by RT‐qPCR (Figure 7C). Accordingly, this oligopeptide failed to antagonize AA production and reduce the accumulations of 4‐HNE, MDA, and Fe induced by TAI in *Gpbar1* ^−/−^ mice (Figure 7F–I), leading to a diminished protective effect against IR‐induced intestinal toxicity (Figure 7J, Figure S19). Overall, these findings demonstrate that activation of the TGR5‐SREBP1‐SCD1 axis plays a critical role in mediating the intestinal radioprotection of AT9(C/G).

## DISCUSSION

The pathogenesis of IRIII is associated with dysbiosis of the gut microbiota and ferroptosis of intestinal epithelial cells. In this study, we developed AT9(C/G), an oligopeptide that markedly alleviated IRIII by regulating the gut microbiota and suppressing ferroptosis. LCA, a secondary BA produced via enteric bacterial metabolism, was identified as the metabolite linking the observed improvement in the microbial community and ferroptosis inhibition. Clinical investigation revealed a negative correlation between IRIII severity and the fecal LCA content, which was corroborated by the positive correlation between the LCA abundance and both villus length and ileal crypt number in AT9(C/G)‐treated mice after TAI. TGR5 is an important G protein‐coupled receptor that mediates the ability of BAs to modulate glucose and lipid metabolism [46]. Our in vitro and in vivo assays confirmed that LCA could reduce cell vulnerability to lipid peroxidation caused by IR via the TGR5‐SREBP1‐SCD1 axis, thereby repressing ferroptosis to achieve intestinal radioprotection.

Primary BAs undergo bacterial conversion to secondary BAs through the action of BSH‐expressing gut microbiota [22, 23]. Our study revealed that AT9(C/G) treatment significantly increased both LCA levels and *B. pseudolongum* abundance in mice. Integrated multiomics analysis demonstrated a positive correlation between intestinal LCA concentration and *B. pseudolongum* enrichment. This relationship was further supported by the finding that LCA levels were increased following *B. pseudolongum* colonization. Pharmacological inhibition of BSH abrogated the increase in LCA abundance induced by *B. pseudolongum*, which confirms that BSH is present in this probiotic. With respect to the enrichment of *B. pseudolongum* caused by AT9(C/G), we suggest that AT9(C/G) can directly target the probiotic and promote its growth, possibly through the potential role of oligopeptides as nitrogen sources or as promoters of polypeptide transport systems that increase bacterial proliferation [47].

In contrast to previous reports identifying LCA as an antineoplastic metabolite that induces ferroptosis [48], our study demonstrated that LCA suppresses IR‐induced ferroptosis in the intestinal epithelium. This functional discrepancy may be explained by tumor‐specific metabolic reprogramming and distinct features of the tumor microenvironment. Consistent with the anti‐ferroptotic activity of LCA, a recent study showed that taurolithocholic acid (TLCA), another secondary BA, also inhibits ferroptosis and alleviates viral hemorrhagic fever [43]. Notably, both LCA and TLCA exert their ferroptosis‐inhibitory effects through TGR5 activation. The tissue‐specific outcomes of TGR5 signaling deserve particular emphasis. Although TGR5 activation in the liver exacerbates lipotoxicity‐induced cell death and inflammation [49], its stimulation in the intestine promotes epithelial regeneration [50]. These divergent effects likely reflect differences in TGR5 expression levels and tissue‐specific metabolic demands [26]. As the principal site of dietary fat absorption, the intestine maintains tightly regulated lipid homeostasis and exhibits high TGR5 expression. Although TGR5 activation is known to attenuate lipid accumulation [46], its role in modulating intestinal ferroptosis remained unclear. Our study establishes a definitive link between TGR5 signaling and the ferroptosis regulatory network. These findings also have broader implications for intestinal pathophysiology. Given that ferroptosis contributes significantly to intestinal ischemia/reperfusion (I/R) injury [51], which can be mitigated by TGR5 activation [52], we propose TGR5‐mediated ferroptosis inhibition as a potential mechanism underlying this protection.

Fatty acid desaturase catalyzes the insertion of carbon–carbon double bonds into the fatty acyl chain of SFAs. SCD1 mediates the initial desaturation step to produce MUFAs [53]. We identified SCD1 as the key downstream effector of TGR5 in mediating the anti‐ferroptotic effects of LCA. Mechanistically, TGR5 activation by LCA promotes the expression of SREBP1, which transcriptionally upregulates SCD1 by targeting its promoter sequence. This increases the MUFA‐PL levels in irradiated cells, counteracting ferroptosis by lowering the PUFA‐PL ratio to mitigate lipid peroxidation and decreasing the intracellular Fe²⁺ level. Notably, BMP 18:1_18:1, a lysosome‐enriched MUFA‐PL that is elevated upon SCD1 induction, was found to mitigate IR‐induced Fe²⁺ accumulation. Such an inhibitory effect is attributed to its ability to attenuate lysosomal membrane lipid peroxidation, thereby preventing the leakage of stored Fe²⁺ [37]. These findings not only highlight the critical role of the SCD1‐MUFA axis in regulating ferroptosis but also support the therapeutic potential of SCD1 inhibition in ferroptosis‐resistant tumors [44]. In addition to upregulating SCD1, our results show that LCA modestly downregulates ACSL4, a key driver of proferroptotic PUFA‐PL synthesis [3]. This coordinated regulation is likely mediated by AKT activation downstream of LCA‐primed TGR5 signaling [54], thereby functioning in concert with SCD1 to suppress IR‐induced ferroptosis.

Since LCA upregulates SCD1 through TGR5 signaling, reduced LCA levels may lead to decreased epithelial SCD1 expression. However, due to an insufficient number of clinical samples, we were unable to evaluate the diagnostic value of SCD1 in patient rectal tissue, as was done for LCA. This remains an important issue for future investigation. Additionally, our study has another limitation. Because AT9(C/G) offers advantages in radioprotection, cost‐effectiveness, and storage stability, we investigated its mechanism of action but did not explore the specific reasons for its superiority over HD5 or other fragments. Future studies should examine differences in microbial composition and GMMs, which may account for the enhanced efficacy of AT9(C/G).

## CONCLUSION

In summary, our findings reveal that AT9(C/G), an oligopeptide derived from the human intestinal defensin HD5, mitigates IR‐induced intestinal toxicity by enriching *B. pseudolongum* and increasing enteric LCA levels. IR induces the accumulation of PUFA‐PL and triggers ferroptosis in intestinal epithelial cells. LCA counteracts this process by reprogramming lipid metabolism through TGR5 activation and subsequent induction of SREBP1 expression, which transcriptionally upregulates SCD1 to catalyze the production of MUFAs. Due to the antagonistic effect of MUFAs against PUFA‐PL formation, LCA reduces ferroptosis after IR and alleviates IRIII. Given the current lack of effective IRIII treatments, AT9(C/G) represents a promising candidate for intestinal radioprotection. Furthermore, because LCA deficiency is involved in the pathogenesis of many diseases, such as inflammatory bowel disease and thrombotic disease [55, 56], and because *B. pseudolongum* supplementation can alleviate Alzheimer's disease [33], AT9(C/G) may function as a peptide prebiotic with pleiotropic health benefits beyond IRIII mitigation.

## METHODS

### Peptide preparation

HD5 was obtained from MCE (HY‐P5694, Shanghai, China). HD5‐derived fragments and FITC‐AT9(C/G) were prepared via automatic solid‐phase synthesis at Guoping Pharmaceutical (Hefei, China). The purity of the products was analyzed using a high‐performance liquid chromatography (HPLC) system (LC‐10AT, SHIMADZU, Kyoto, Japan) with an Inertsil ODS‐SP column (5 μm, 4.6 × 250 mm). Separation was achieved using a linear gradient of 15–55% buffer B (0.1% trifluoroacetic acid in acetonitrile) in buffer A (0.1% trifluoroacetic acid in water). The peptide molecular weights were determined using a SHIMADZU LCMS‐2020 mass spectrometer. HPLC and mass spectrum of synthetic peptides are shown in Figure S20. The observed molecular weights of these peptides are close to their theoretical values.

### TAI mouse experiment

Six‐ to eight‐week‐old C57BL/6J mice (body weight: 18–20 g) were procured from Vital River Laboratory Animal Technology Co., Ltd (Beijing, China). The mice were maintained in a specific pathogen‐free facility at the Institute of Combined Injury, PLA, Army Medical University (AMU), under controlled environmental conditions (22 ± 2°C, 40–70% relative humidity) with a 12‐h light/dark cycle. The male mice were randomly divided into eight groups: the Sham, IR, HD5 + IR, AT9 + IR, AT9(C/G) + IR, AT28 + IR, TG26 + IR, and CA23 + IR groups. Fifty female mice were randomly assigned to five groups: the Sham, IR, HD5 + IR, AT9 + IR, and AT9(C/G) + IR groups. All mice received daily oral administration of 35 µM peptide solutions (200 µL in PBS) for seven consecutive days (q.d.). For TAI, the mice were anesthetized with 400 µL of 1.25% tribromoethanol and exposed to 12 Gy γ‐radiation from a ^60^Co source (dose rate: 0.4 Gy/min) at zeitgeber time 1. Peptide treatment continued for an additional 3 days post‐TAI.

To investigate the role of the gut microbiota in AT9(C/G)‐mediated radioprotection, mice were randomly assigned to six experimental groups: the Sham, IR, ABX + IR, AT9(C/G) + IR, AT9(C/G) + IR + ABX, and LCA + IR + ABX groups. An antibiotic cocktail consisting of ampicillin (1 mg/mL; A600064‐0025, BBI Life Science, Shanghai, China), metronidazole (1 mg/mL; A6000633‐0025, BBI Life Science), neomycin (0.5 mg/mL; A610366‐0025, BBI Life Science), and vancomycin (0.5 mg/mL; V105495, Aladdin, Shanghai, China) was added to the drinking water 1 week prior to AT9(C/G) treatment. For the LCA supplementation studies, the 60 mice were divided into six groups: the Sham, IR, and four LCA‐treated IR groups (5, 10, 15, and 20 mg/kg). LCA (HY‐B0172, MCE) was administered by oral gavage for seven consecutive days (q.d.), with TAI performed 4 days after LCA initiation. Mouse survival was then monitored for 30 days, and the DAI score was assessed on Day 4 post‐TAI using established criteria (Table S4) [34]. All the animal procedures were approved by the AMU Institutional Review Board (Approval No. AMUWEC20230292).

### Histopathologic analysis

Mice were allocated into 16 groups (the Sham, IR, HD5 + IR, AT9 + IR, and AT9(C/G) + IR groups; the Sham, IR, AT9(C/G), AT9(C/G) + IR, AT9(C/G) + IR + MF‐438, and AT9(C/G) + IR + *Gpbar1* ^−/−^ groups; the Sham, IR, LCA, LCA + IR, and LCA + IR + *Gpbar1* ^−/−^ groups) to perform the TAI experiment. Prior to TAI induction, mice were orally administered peptides for 7 days or LCA (15 mg/kg) for 4 days (q.d.), with treatments continuing for 3 days post‐TAI. MF‐438 (HY‐15822, MCE) was administered at 30 mg/kg/day via oral gavage. *Gpbar1* ^−/−^ mice (strain no. T012754), which were purchased from GemPharmatech (Nanjing, China), were genotyped with the results shown in Figure S21. The mice were euthanized via tribromoethanol overdose 3 days after TAI, and small intestine tissues were surgically collected. Ileum samples were fixed in Carnoy's fluid (G1120, Servicebio, Wuhan, China) at 4°C for 48 h, dehydrated through an ethanol gradient, and paraffin‐embedded. Sections (3 µm thick) were prepared for H&E staining, followed by the determination of ileal villus height and crypt number. Morphometric analysis was performed in a blinded manner by two investigators. Ten straight, well‐oriented villi (with their axis perpendicular to the muscularis mucosae) were selected per specimen, and their height was measured from the apex to the crypt–villus junction along the central axis using ImageJ (v1.53). For crypt density, the muscularis mucosae was outlined with a calibrated line in three randomly selected, non‐overlapping fields. Crypt density was calculated as the total count divided by the total line length for each specimen.

### Evaluation of endotoxemia

Fresh blood samples from the irradiated mice were centrifuged (3000 rpm, 15 min) to isolate serum. A 100 µL aliquot of serum was mixed with an equal volume of Tachypleus amebocyte lysate (EC80545, Xiamen Bioendo Technology, China) and incubated at 37°C for 10 min. After the addition of 100 µL of chromogenic substrate and a 6‐min incubation, the reaction was terminated with hydrochloric acid. The absorbance at 545 nm was measured using an M2e microplate reader (Molecular Devices, San Jose, CA) after the addition of the azo reagent. Serum levels of IL‐6 and IL‐1β were quantified via enzyme‐linked immunosorbent assay (ELISA) using Elabscience (Wuhan, China) kits (E‐EL‐M0044‐96T and E‐EL‐M0037‐96T, respectively).

### Biocompatibility evaluation

Six‐ to eight‐week‐old C57BL/6J mice weighing 18–20 g were randomly divided into two groups (*n* = 10 mice per group). AT9(C/G) was administered orally at a dose of 35 µM in 200 µL, once daily for 1 month. The sham group was treated with sterile PBS. An automatic biochemical analyzer (DS161, SINNOWA, Nanjing, China) was applied to determine the alanine aminotransferase, aspartate aminotransferase, creatinine, and urea levels in mouse blood obtained on Day 30 after peptide treatment. Mouse blood obtained through the tail vein was analyzed on a XT‐2000i fully automatic hematology analyzer (Sysmex, Kobe, Japan). The mice were then sacrificed to obtain the intestine, heart, liver, spleen, lungs, and kidneys, which were processed for H&E staining. A DX51 optical microscope (Olympus, Tokyo, Japan) was used to observe the pathological changes.

### Fecal microbiota transplantation (FMT)

Fecal samples from AT9(C/G)‐treated mice were collected after 7 days, homogenized in sterile PBS (1 g/10 mL), and centrifuged (3000 rpm, 30 s). The supernatant was further centrifuged (12,000 rpm, 4°C, 5 min) to pellet the microbiota, which was subsequently resuspended in 2.5 mL of PBS for oral gavage (q.d.). Recipient mice received a 7‐day ABX pretreatment to deplete the gut microbiota prior to FMT. After 7 days of daily FMT, the mice underwent TAI, followed by an additional 3 days of FMT. As a control, the fecal microbiota from healthy wild‐type mice was processed identically. Mouse survival was recorded as previously described for the TAI model.

### Metagenomic analysis

Fresh fecal samples were collected from mice treated with PBS or AT9(C/G) for 7 days and stored at −80°C until processing. Samples were also collected from the following two cohorts: mice subjected to sham or AT9(C/G) treatment 3 days after TAI; and patients before and 7 days after SCRT. Fecal DNA was extracted with a magnetic soil and stool DNA kit (4992736, TIANGEN, Beijing, China) and fragmented into short sequences. Prior to size selection, the DNA fragments were end‐repaired, poly(A)‐tailed, and ligated with Illumina sequencing adapters. Library quality was assessed using an Agilent 2100 Bioanalyzer (Agilent Technologies, Beijing, China), and library quantification was performed via qPCR (effective concentration > 3 nM). Pooled libraries were sequenced on the Illumina PE150 platform (Novogene, Beijing, China).

Raw sequencing data were processed using fastp (v0.23.1) to obtain high‐quality clean reads, which were subsequently aligned to the mouse reference genome (GRCm38) to exclude host‐derived sequences. MEGAHIT (parameters: ‐‐presets meta‐large, ‐‐end‐to‐end, ‐‐sensitive, ‐I 200, ‐X 400) was used for the de novo assembly of the clean data. Open reading frames were predicted from scaftigs (≥500 bp) using MetaGeneMark, and a nonredundant gene catalog was generated via CD‐HIT. For gene abundance analysis, clean reads were mapped to the gene catalog using Bowtie2, and genes with ≤2 reads per sample were discarded to produce the final catalog. Taxonomic annotation was performed by aligning unigenes against the Micro_NR database using DIAMOND, with species assignments determined with the LCA algorithm. Microbial α diversity was assessed using seven indices: Observed species, ACE index, the Shannon index, the Simpson index, the Chao1 index, and Good's coverage through the NovoMagic analytical platform. For β‐diversity evaluation, ANOSIM was conducted based on Bray–Curtis distance to assess significant differences. Taxonomic differences between groups were identified via LEfSe analysis with a linear discriminant analysis (LDA) score cutoff of 3.

### Bacterial culture and determination

*E. coli* (strain no. 25922), *B. pseudolongum* (strain no. 25526), and *C. butyricum* (strain no. 19398), which were obtained from the American Type Culture Collection (ATCC), were cultured in Mueller‐Hinton broth (M8556, Solarbio, Beijing, China), modified MRS broth (373873, BeNa Culture Collection, Xinyang, China), and reinforced clostridial medium (BZ2507105, Boer, Chongqing, China), respectively. *C. symbiosum* (strain no. 364164), *P. copri* (strain no. 337399), and *A. rava* (strain no. 358370) were purchased from BeNa Culture Collection (BNCC). *P. copri* and *A. rava* were cultured in modified PYG broth (BNCC363892). Bacteria were cultured in an anaerobic workstation filled with 80% N₂, 10% CO₂, and 10% H₂. Each bacterial suspension was diluted to 8 × 10^8^ CFU/mL and coincubated with 35 μM AT9(C/G) in a 48‐well plate for 16 h. The initial concentration of *E. coli* was prepared at 1 × 10^8^ CFU/mL. The optical density (OD_600_) of the bacterial suspension was measured using an M2e microplate reader (Molecular Devices). These experiments were performed with three biological replicates.

### Biolayer interferometry (BLI)

*B. pseudolongum* (1 × 10^8^ CFU/mL, 500 µL) was incubated with 100 µM biotin (Genemore, Shanghai, China) in the dark for 30 min. After incubation, the bacteria were harvested by centrifugation (10,000 rpm, 4°C, 5 min) and washed twice with sterile PBS to remove unbound biotin. The biotinylated bacteria were then immobilized onto streptavidin biosensors. The association of increasing concentrations (25, 50, 100, and 200 µM) of AT9(C/G) was monitored using BLI (Octet Red 96, Sartorius, Göttingen, Germany) for 180 s in PBS, with constant shaking at 1000 rpm. All data were corrected for the background signal from the 0 µM peptide control using ForteBio Data Analysis 7.0 software.

### Targeted metabolomic analysis

Fecal samples (5 mg each) from PBS‐ or AT9(C/G)‐treated mice (*n* = 6 mice per group) were homogenized for 3 min using zirconium oxide beads. Metabolites were extracted by adding 120 μL of methanol (CH_3_OH) containing internal standards, followed by another 3 min of homogenization and centrifugation (18,000 *g*, 20 min). A 20 μL aliquot of the supernatant was transferred to a 96‐well plate for derivatization on an epMotion Workstation (Eppendorf, Hamburg, Germany). The samples were diluted with 330 μL of ice‐cold 50% CH_3_OH, incubated at −20°C for 20 min, and centrifuged (4000 *g*, 4°C, 30 min). The resulting supernatant (135 μL) was combined with 10 μL of internal standards in a new plate for LC‐MS analysis.

Targeted metabolomics analysis was performed using a UPLC‐MS/MS system (ACQUITY UPLC–Xevo TQ‐S, Waters, Milford, MA) at Metabo‐Profile (Shanghai, China). Separation was achieved using a BEH C18 column (1.7 μm, 2.1 × 100 mm) with an 18‐min linear gradient (0.4 mL/min flow rate). The mobile phases consisted of 0.1% aqueous formic acid (eluent A) and acetonitrile/isopropanol (7:3, eluent B). MS detection utilized positive/negative polarity switching with the following parameters: capillary voltage of 1.5 kV (ESI+) or 2.0 kV (ESI−), source temperature of 150°C, desolvation temperature of 550°C, and desolvation gas flow rate of 1000 L/h. Test mixtures, internal standards, and pooled samples served as quality controls. The raw data were processed using MassLynx (v4.1; Waters) for peak integration, calibration, and quantification. Statistical analysis was performed on the iMAP platform (v1.0; Metabo‐Profile), with metabolites showing *p* < 0.05, VIP > 1, and FC > 2 or < 0.5 considered significantly altered. The correlation between the metabolomic and microbiota data was analyzed using Spearman's method in R.

### Fecal and serous LCA determination

Fecal samples were collected 3 days after TAI from mice treated with or without AT9(C/G). After being weighed, the samples were homogenized in 1 mL of PBS using magnetic beads for 3 min. The homogenate was centrifuged at 3000 rpm for 15 min at 4°C to obtain the supernatant. To assess the fecal LCA levels following *B. pseudolongum* colonization, the ATCC strain (no. 25526) was anaerobically cultured in MRS broth at 37°C. Bacterial suspensions were prepared in sterile PBS, and the mice received 2 × 10^8^ CFU of *B. pseudolongum* (200 µL) via oral gavage. GR‐7 (10 mg/kg; HY‐135747, MCE) was administered as previously described [23]. Following 7 days of treatment, fecal samples were collected and processed as described above. For the determination of LCA in mouse serum, a 20 μL aliquot of each sample was placed in a 96‐well plate. Then, 120 μL of ice‐cold CH_3_OH containing internal standards was added, and the mixture was vortexed vigorously for 5 min. After centrifugation at 4°C (4000 *g*, 30 min) and derivatization, the plate was then sealed and incubated at 30°C for 60 min. The reaction was quenched by dilution with 330 μL of ice‐cold 50% CH_3_OH. Following a second centrifugation under identical conditions, 135 μL of the supernatant was transferred to a new 96‐well plate. Finally, 10 μL of internal standard was added to each well. The LCA levels were determined with a UPLC–MS/MS system (ACQUITY UPLC–Xevo TQ–S, Waters).

### Cell line authentication and culture

The HIEC‐6 (Cat# CRL‐3266; RRID: CVCL_6C21), IEC‐6 (Cat# CRL‐1592; RRID: CVCL_0343), and HCT116 (Cat# CCL‐247; RRID: CVCL_0291) cell lines were obtained from ATCC in September 2024, August 2023, and July 2023, respectively. These cell lines have not been previously reported as misidentified or contaminated. The cells were routinely tested negative for mycoplasma contamination using the GTB Mycoplasma Detection Kit (Genetic Testing Biotechnology, Suzhou, China). The HIEC‐6 and HCT116 cell lines were authenticated by short tandem repeat (STR) profiling, with each showing a 100% match to their respective reference profiles. Owing to the lack of standardized STR profiling protocols and public databases for rat‐derived cells, we confirmed the species origin of the IEC‐6 cell line by species‐specific PCR targeting the mitochondrial cytochrome c oxidase subunit 1. The cells were maintained in DMEM (Thermo Fisher Scientific, Shanghai, China) supplemented with 10% FBS, cultured at 37°C under 5% CO₂, and used within eight passages.

### Cell experiments

HIEC‐6 and IEC‐6 cells were seeded in sterile 96‐well plates at 5000 cells/well and allowed to adhere overnight. Cells were exposed to 8 Gy γ‐rays using a ^60^Co source (dose rate: 0.4 Gy/min) [34]. Prior to irradiation, LCA, bDCA (ZS‐20121, ZZBIO, Shanghai, China), and valerylcarnitine (HY‐113266, MCE) were added at final concentrations of 5, 10, or 20 µM, respectively, 6 h before IR. Erastin (5 µM; HY‐15763, MCE) was introduced 4 h before LCA treatment. To investigate the role of SCD1, FADS1, and FADS2 in LCA‐mediated radioprotection, we silenced these genes in IEC‐6 cells by transfection with specific siRNAs (Table S5) using the HiPerFect transfection reagent (301705, QIAGEN, Shanghai, China). To further dissect the lipid metabolism involvement, we treated IEC‐6 cells with 50 µM BMP 18:1_18:1 (HY‐N16011, MCE) or 12.5 µM OA (HY‐N1446, MCE) for 6 h prior to irradiation and assessed the impact on IR‐induced ferroptotic hallmarks. In parallel, the effect of LCA on the survival of irradiated tumor cells was evaluated in HCT116 cells, which were treated with 10 and 20 μM LCA for 6 h before irradiation. Cell viability was evaluated 24 h post‐IR using a CCK‐8 assay (CK04, Dojindo, Shanghai, China). The morphological changes of mitochondria in cells were observed using a JEM‐1400 FLASH transmission electron microscope (Japan Electron Optics Laboratory, Tokyo). Lipid peroxidation was assessed using the fluorescent probe C11‐BODIPY581/591 (D3861, Thermo Fisher Scientific). Intracellular Fe²⁺ levels were measured with 10 μM FerroOrange (F374, Dojindo), and fluorescence intensity was quantified with a FACSVerse™ flow cytometer (BD, Franklin Lakes, NJ). The total iron content was determined colorimetrically (I291, Dojindo). 4‐HNE and MDA levels were evaluated using ELISA (D751041, Sangon Biotech, Shanghai, China) and the TBA method (M496, Dojindo), respectively. Lysosomes were stained with LysoTracker Red (C1046, Beyotime, Shanghai, China), and lipid peroxidation was measured using Liperfluo (L248, Dojindo). These assays were performed with six biological replicates.

### Human intestinal organoid assay

Normal terminal ileum tissues were obtained from patients during colonoscopy, in accordance with the guidelines of the Ethics Committee of the Army Medical Center of the PLA (Approval No. 2022‐223‐01). For crypt isolation, the ileum tissue was digested with MasterAim® Organoid Cluster Dissociation Solution (100‐358, AimingMed, Hangzhou, China) at room temperature for 20 min and monitored carefully until crypts appeared softened but intact. Cold Dulbecco's PBS supplemented with 1% penicillin‐streptomycin (DPBS‐PS) was then added to terminate digestion, and the mixture was centrifuged at 200 *g* for 3 min. The pellet was resuspended in DPBS‐PS and filtered through a 70 μm cell strainer to obtain crypt‐enriched filtrate. Intestinal crypts were further gently mixed with Matrigel® (356231, Corning, Shanghai, China) at a density of 3–4 crypts/μL. Forty aliquots of the crypts were seeded as central domes in a 48‐well plate and allowed to solidify for 15 min at 37°C. Pre‐warmed Human Small Intestinal Organoid Complete Medium (10‐100‐352, AimingMed) was finally added, and the culture was conducted at 37°C in a 5% CO₂ atmosphere. Immunofluorescence was performed on the collected organoids to measure the expression of human lysozyme, mucin 2, and Ki‐67, verifying their human origin (Figure S5). LCA was administered at final concentrations of 5, 10, and 20 μM, respectively, 6 h prior to IR. Organoid viability was assessed by capturing bright‐field images on 72 h post‐IR. The survival proportion was determined by counting intact and viable HSIOs using ImageJ (v1.53), with data normalized to non‐irradiated controls. Following 48 h of treatment with or without 10 μM LCA, irradiated HSIOs were also processed for the determination of Fe²⁺, MDA, and 4‐HNE levels.

### In‐house patient and sample collection

Human fecal and serum samples were collected from 20 rectal cancer patients receiving neoadjuvant SCRT combined with chemoimmunotherapy at Daping Hospital, China (December 2024–June 2025). The study was conducted in accordance with the guidelines of the Ethics Committee of the Army Medical Center of the PLA (Approval No. 2024‐285‐01), and written informed consent was obtained from all participants, who retained the right to withdraw at any time. Detailed patient characteristics are provided in Table S6. Data on socioeconomic status, ethnicity, race, or ancestry were not collected. All participants received the same SCRT regimen (25 Gy in 5 fractions of 5 Gy each, delivered over five consecutive days) during the initial phase of treatment. After a 1‐week rest period following SCRT, patients proceeded to two cycles of combined CAPOX chemotherapy and ivonescimab administration at 3‐week intervals. Fecal and peripheral blood samples were collected at baseline (pre‐SCRT) and on Day 7 post‐SCRT for LCA and IL‐6 quantification, respectively. Fecal samples were immediately stored at −80°C in sterile containers after collection. Serum IL‐6 levels were measured using an Elecsys IL‐6 kit (09015604190, Roche, Basel, Switzerland) on the Roche Cobas e601 fully automated electrochemiluminescence immunoassay analyzer.

### Proteomic analysis

Proteins were extracted from IEC‐6 cells treated with or without 10 µM LCA 24 h post‐IR using lysis buffer supplemented with 8 M urea and 100 mM triethylammonium bicarbonate (TEAB; T7408, Sigma‐Aldrich, Shanghai, China). After centrifugation at 12,000 *g*, the supernatant was collected and reduced with 1 M dithiothreitol (DTT; D9163, Sigma‐Aldrich) at 56°C for 1 h, followed by a 2‐min ice bath. Alkylation was performed in the dark at room temperature for 1 h using anhydrous iodoacetamide (I6125, Sigma‐Aldrich). Protein quantification was followed by tryptic digestion, and the pH was adjusted to <3.0 with formic acid (FA). Peptides were desalted, washed three times with 0.1% FA/3% acetonitrile (ACN), and eluted with 0.1% FA/70% ACN. The samples were then analyzed using a Vanquish Neo UHPLC system (Thermo Fisher Scientific) coupled with a C18 precolumn (5 mm × 300 µm, 5 µm) and an analytical column (150 µm × 15 cm, 2 µm). Mass spectrometry was performed at Novogene on a Thermo Orbitrap Astral instrument with an easy spray ion source (1.9 kV spray voltage). Data were acquired in data‐dependent mode with the following parameters: primary MS, scan range: 380–980 (m/z), resolution: 240,000 (200 m/z); secondary MS, scan range: 150–2000 (m/z). Raw data were processed using DIA‐NN software, after which the peptides and proteins with FDR values > 1% were filtered out. Differentially expressed proteins were defined as those with *p* < 0.05 and FC > 1.5 or FC < 0.67. Functional enrichment analysis was conducted using the KEGG and InterPro databases.

### Lipidomic analysis

Lipids were extracted from 2 × 10^6^ IEC‐6 cells (Con, IR, LCA + IR, and LCA + IR + Si‐*Scd1*) using a modified Bligh and Dyer method as described previously [57]. The cells were homogenized in 750 µL of chloroform (CHCl_3_):CH_3_OH:water (3:6:1, v/v/v) and incubated at 4°C for 1 h. Phase separation was induced by adding 350 µL of deionized water and 250 µL of CHCl_3_, followed by centrifugation to collect the lower organic phase. The extraction was repeated with 450 µL of CHCl_3_, and the combined lipid extracts were dried using a SpeedVac (Thermo Fisher Scientific) and stored at −80°C. The remaining aqueous phase and cell pellet were dried separately in a SpeedVac (H₂O mode). Total protein content was determined from the dried pellet using the Pierce BCA Protein Assay Kit (23227, Thermo Fisher Scientific).

Lipid profiling was performed at LipidALL Technologies (Changzhou, China) using a Shimadzu Nexera 20AD‐HPLC system coupled with a QTRAP 6500 PLUS mass spectrometer (Sciex, Concord, ON, Canada). Polar lipids were separated via normal‐phase HPLC on a TUP‐HB silica column (150 mm × 2.1 mm, 3 µm) with the following mobile phases: phase A, CHCl_3_:CH_3_OH:NH_4_OH (89.5:10:0.5); and phase B, CHCl_3_:CH_3_OH:NH_4_OH:water (55:39:0.5:5.5). Lipid quantification was achieved using multiple reaction monitoring with the following internal standards: phospholipids: d9‐PC32:0, d9‐PC36:1p, d7‐PE33:1, d9‐PE36:1p, d31‐PS, d7‐PA33:1, d7‐PG33:1, and d7‐PI33:1 (Avanti Polar Lipids, Birmingham, AL); and free fatty acids: d31‐16:0 (Sigma‐Aldrich) and d8‐20:4 (Cayman Chemicals, Ann Arbor, MI).

### Confocal microscopy

*B. pseudolongum* (1 × 10^8^ CFU in 1 mL) was sequentially stained with FM4−64 (5 µg/mL; T13320, Invitrogen, Thermo Fisher Scientific) and Hoechst 33342 (2 µM; 62249, Thermo Fisher Scientific). After staining, the bacteria were collected by centrifugation at 4°C, resuspended in 100 µL of sterile PBS, and coincubated with 100 µg/mL FITC‐AT9(C/G) at 37°C for 30 min. A 10 µL aliquot of the mixture was then spread on a slide and fixed with 4% paraformaldehyde. For immunofluorescence staining, the paraffin sections were deparaffinized with dimethylbenzene and ethyl alcohol and subjected to antigen retrieval in citrate buffer (10 mM, pH 6.0) at 95°C−100°C for 15 min. To observe the expression levels of SCD1 and SREBP1 in IEC‐6 cells, the cells were cultured on sterile glass slides in a 12‐well plate (2 × 10^5^ cells/well). After treatment with IR or LCA, the cells were fixed with 4% paraformaldehyde for 10 min. The samples were subsequently incubated with antibodies for SCD1 and SREBP1 detection. Detailed information on the antibodies used in this study is shown in Table S7. Images were captured with a Zeiss LSM 780 NLO confocal microscope. Quantitative analysis of fluorescence intensity was conducted with ZEN 2011 (blue edition).

### RT–qPCR

RNA was extracted from mouse ileum tissues and IEC‐6 cells using a RNAiso Plus reagent (9109, TaKaRa, Dalian, China). The RNA was reverse‐transcribed using a ABScript Neo RT Master Mix for qPCR with a gDNA remover kit (RK20433, ABclonal, Wuhan, China). The mRNA expression was analyzed using 2 × Universal SYBR Green Fast qPCR Mix (RK21203, ABclonal) with a CFX96 Touch™ Real‐Time PCR detection system (Bio‐Rad, Hercules, CA). β‐Actin or GAPDH was used for normalization. The primers used are presented in Table S8. The data were analyzed using a Bio‐Rad iQ5 standard‐edition optical system (v2.1). This assay was independently repeated six times.

### RNA‐seq

Ileal tissues from mice treated with or without AT9(C/G) (*n* = 3 mice for each group) were collected 3 days after TAI for total RNA extraction using TaKaRa RNAiso Plus reagent. Subsequent RNA sequencing and library preparation were performed by Novogene. Briefly, mRNA was isolated using poly‐T oligo‐attached magnetic beads, followed by first‐strand cDNA synthesis with random hexamer primers and M‐MuLV reverse transcriptase. Second‐strand cDNA was generated using DNA polymerase I and dNTPs, and the library fragments were subsequently purified with the AMPure XP system (Beckman Coulter, Brea, CA). An Illumina NovaSeq. 6000 platform was used for sequencing. Differentially expressed genes were identified using DESeq. 2 (v1.4.5), with significance thresholds set at *p* < 0.05 and FC > 1.5 or FC < 0.67. Functional enrichment analysis was performed on the basis of the GO database.

### Immunoblotting assay

IEC‐6 cells and mouse ileal tissues were collected and lysed for protein extraction. Twenty‐five micrograms of each sample was resolved via 10% sodium dodecyl sulfate‐polyacrylamide gel electrophoresis. Detailed information concerning the antibodies used to detect TGR5, SCD1, SREBP1, p‐AKT, and AKT is shown in Table S9. A BeyoECL Plus chemiluminescence kit (P0018S, Beyotime) and a Bio‐Rad ChemiDoc^TM^ MP Imaging System were used to visualize the protein bands. β‐Actin was employed as a reference.

### Streptavidin‐bead pull‐down assay

Cells were treated with 10 µg/mL biotinylated‐LCA (R‐QT‐0530, Xi'an Ruixi Biotechnology, China) or biotinylated‐IgG (P01857‐1, MCE) for 1 h at 37°C under 5% CO₂. After three washes with ice‐cold Tris‐buffered saline, the cells were lysed in 500 µL of RIPA buffer (P0013K, Beyotime) supplemented with protease inhibitors. The lysates were then incubated overnight at 4°C with BeyoMag Streptavidin Magnetic Beads (P2151, Beyotime) to capture the bead−antigen complexes. After three washes with NETN buffer (20 mM Tris‐HCl, 100 mM NaCl, 1 mM EDTA, 0.5% Nonidet P‐40), the complexes were eluted by boiling in 15 μL of 2 × loading buffer (P0015B, Beyotime) for 5 min and subsequently analyzed by immunoblotting.

### Dual‐luciferase reporter assay

A series of 5' truncation fragments of the *SCD1* promoter region, spanning positions −2000 to +200, −1800 to +200, −1500 to +200, −1000 to +200, and −500 to +200, were each amplified and cloned into the HindIII site of the pGL3‐basic vector (Promega, Beijing, China). The resulting constructs were designated pGL3‐SCD1‐P1 to P5, respectively. Additionally, a mutant reporter plasmid (pGL3‐SCD1‐MUT) was generated from pGL3‐SCD1‐P2 by replacing the SREBP1 binding motif (ATCACACCAC) with a single adenine base using a site‐directed mutagenesis kit (TaKaRa MutanBEST). For luciferase reporter assays, each recombinant reporter plasmid was cotransfected with the pRL‐TK internal control vector (Promega) into HIEC‐6 cells using Lipofectamine 2000 (11668019, Thermo Fisher Scientific) in OptiMEM medium (Hyclone, Thermo Fisher Scientific). Twenty‐four hours after transfection, cells were subjected to either the control treatment or SREBP1 overexpression for an additional 24 h. Luciferase activities were measured using the Dual‐Luciferase Reporter Assay System (E1910, Promega). Firefly luciferase signals were normalized to the corresponding Renilla luciferase activity to control for transfection efficiency.

### ChIP assay

After 24 h of treatment, either control or SREBP1 overexpression, HIEC‐6 cells were fixed with 1% formaldehyde for 10 min. The cells were then lysed in SDS lysis buffer, and the chromatin was sonicated to shear the DNA into fragments ranging from 200 to 1000 bp. Immunoprecipitation was performed using 2 μg of anti‐SREBP1 antibody (14088‐1‐AP, Proteintech, Wuhan, China), with normal IgG serving as the negative control. The immunoprecipitated DNA was analyzed by PCR using primers spanning the region −1805 to −1708, which covers the putative SREBP1 binding site (−1737 to −1728). A primer set targeting a distal region without SREBP1 binding sites (−359 to −190) was used as a genomic negative control, while the total input DNA was included as a positive control. All ChIP primer sequences are provided in Table S10.

### Determination of arachidonic acid and oleic acid in the mouse ileum

Ileal tissue samples from mice were weighed and homogenized in 200 μL of 0.9% NaCl. The homogenate was extracted twice with 1 mL of CHCl_3_:CH_3_OH (2:1, v/v), and the combined organic phase was evaporated under a gentle nitrogen stream. Subsequently, 1 mL of 2.5% sulfuric acid in CH_3_OH was added, and the mixture was hydrolyzed at 80°C in a water bath for 1 h. After cooling to room temperature, 1 mL of n‐hexane was added, and the solution was vortexed thoroughly. The upper n‐hexane layer was carefully transferred to a 1.5 mL microcentrifuge tube, concentrated under nitrogen, and adjusted to a final volume of 100 μL. Chromatographic analysis was performed using an Agilent 6890 N gas chromatograph equipped with a WM‐CN100 capillary column (60 m × 0.25 mm × 0.2 μm).

### Statistical analysis

Statistical significance was determined using one‐way analysis of variance (ANOVA) followed by Tukey's multiple comparison test in GraphPad Prism (version 10.1.2). Changes in fecal LCA levels in patients before and after SCRT were analyzed using two‐tailed paired *t*‐test, while alterations in gut microbial diversity (ACE and Shannon indices) were assessed with two‐tailed unpaired *t*‐test. Survival was analyzed with the Mantel–Cox log‐rank test. The Kruskal−Wallis test was used to analyze DAI scores and fecal LCA levels after *B. pseudolongum* supplementation. The correlation between fecal LCA content and ileal villus length or crypt number in AT9(C/G)‐treated mice after TAI, as well as the correlation between fecal LCA content and serum IL‐6 level in patients following SCRT, was determined by Spearman correlation analysis. Statistical comparisons of the expression levels of three fatty acid desaturases, derived from proteomics data between control and LCA‐treated irradiated cells, were performed using the non‐parametric Mann−Whitney *U* test. Statistical parameters were represented in the figure legends. A *p*‐value < 0.05 was considered to indicate statistical significance.

## AUTHOR CONTRIBUTIONS

**Xue Ouyang**: Data curation; investigation; visualization; writing—original draft. **Yingjuan He**: Investigation; methodology. **Zihao Jin**: Investigation; validation. **Shaobo Wang**: Investigation; methodology. **Gaomei Zhao**: Formal analysis; funding acquisition. **Xiaona Su**: Data curation. **Jia Du**: Data curation. **Yin Chen**: Software. **Chengzhang Tan**: Formal analysis. **Xin Li**: Formal analysis. **Songling Han**: Visualization. **Bin Wang**: Resources. **Jinghong Zhao**: Project administration. **Jianqin Niu**: Resources. **Chuan Chen**: Resources; project administration; supervision. **Junping Wang**: Conceptualization; writing—review and editing; supervision. **Cheng Wang**: Conceptualization; funding acquisition; writing—review and editing; writing—original draft. All authors have read the final manuscript and approved it for publication.

## CONFLICT OF INTEREST STATEMENT

The authors declare no conflicts of interest.

## ETHICS STATEMENT

Participants' socioeconomic status, ethnicity, and race/ancestry were not collected. Patient specimens and data were in accordance with the guidelines of the Ethics Committee of the Army Medical Center of the PLA (Approval Nos. 2022‐223‐01 and 2024‐285‐01). All animal experiments were approved by the Army Medical University Institutional Review Board (Approval No. AMUWEC20230292). This study meets our standards of animal ethics.

## Supporting information


**Figure S1.** Survival rates of female mice treated with HD5, AT9, or AT9(C/G) after TAI.
**Figure S2.** Biocompatibility evaluation of a one‐month course of AT9(C/G) in mice.
**Figure S3.** Localization of FITC‐AT9(C/G) in *B. pseudolongum*.
**Figure S4.** Viability of irradiated cells treated with different concentrations of bDCA or valerylcarnitine.
**Figure S5.** Immunofluorescence characterization of human small intestinal organoids.
**Figure S6.** Viability of irradiated HCT116 cells treated with or without LCA.
**Figure S7.** Mitigative effect of LCA on IRIII in mice.
**Figure S8.** Serum LCA levels in irradiated mice with or without AT9(C/G) treatment.
**Figure S9.** The ratio of mean fluorescence intensity between oxidized and non‐oxidized probes.
**Figure S10.** Representative images of HSIOs treated with erastin in the presence or absence of LCA.
**Figure S11.** LCA reprograms lipid metabolism in irradiated cells.
**Figure S12.** Top six functional domains identified through InterPro enrichment analysis.
**Figure S13.** Influence of LCA on the transcription of *Scd1*, *Fads1*, *Fads2*, *Gpx4*, *Nfe2l2*, and *Acsl4* in irradiated cells.
**Figure S14.** Assessment of siRNA‐mediated gene knockdown by RT‐qPCR.
**Figure S15.** Influence of OA supplementation on the adverse effects resulting from *Scd1* silencing on the anti‐ferroptotic activity of LCA.
**Figure S16.** Immunofluorescence microscopy revealing the expressions of SCD1 and SREBP1 in IEC‐6 cells.
**Figure S17.** Maps of five truncated SCD1 reporter plasmids and a SREBP1 overexpression plasmid.
**Figure S18.** Ratios of p‐AKT to AKT in mouse ileum following LCA or AT9(C/G) treatment.
**Figure S19.** Villus length and number of crypts per millimeter in the ileum of irradiated mice.
**Figure S20.** HPLC and MS spectra of synthetic peptides.
**Figure S21.** Genotyping results of *Gpbar1* ^‐/‐^ mice.

**Table S1.** Differentially abundant microbial taxa identified by LEfSe analysis of the gut metagenome.
**Table S2.** Changes in fecal metabolites in mice following AT9(C/G) treatment compared to the sham group.
**Table S3.** JASPAR‐predicted top three SREBP1 binding sites in the *SCD1* promoter.
**Table S4.** The specific criteria for DAI scoring.
**Table S5.** Sequences of siRNAs used in this study.
**Table S6.** Information on oncology patients.
**Table S7.** Information on the antibodies used in immunofluorescence.
**Table S8.** Information on the primers used for RT‐qPCR.
**Table S9.** Information on the antibodies used in immunoblotting.
**Table S10.** Information on the primers used for ChIP assay.

## Data Availability

All data generated or analyzed during this study are included in this published article and its supporting information files. Additional data are available from the corresponding author upon reasonable request. Due to ethical and legal restrictions, individual‐level data from the patients with locally advanced rectal cancer who underwent SCRT cannot be made publicly available. Access to these data is provided upon request to the data manager, Cheng Wang (wangcheng@tmmu.edu.cn), and is subject to local policies and regulations. The request process involves submitting a research proposal to the study management team for approval. Once approved, data analysis must be conducted on a secured local server with controlled access, in compliance with relevant data protection regulations, including GDPR. Raw sequencing data are deposited at the National Genomics Data Center (NGDC) under the accession numbers: PRJCA044489 (Metagenomics, before TAI, mouse fecal samples; https://ngdc.cncb.ac.cn/gsa/browse/CRA029225), PRJCA044490 (Metagenomics, after TAI, mouse fecal samples; https://ngdc.cncb.ac.cn/gsa/browse/CRA029218), PRJCA053109 (Metagenomics, patient fecal samples; https://ngdc.cncb.ac.cn/gsa-human/browse/HRA015356), PRJCA044492 (RNA‐seq, https://ngdc.cncb.ac.cn/gsa/browse/CRA029221), PRJCA044493 (Proteomics, https://ngdc.cncb.ac.cn/omix/release/OMIX011398), and PRJCA044494 (Lipidomics, https://ngdc.cncb.ac.cn/omix/release/OMIX011401). Omics data were visualized via the public platform https://www.bioinformatics.com.cn/. The data and scripts for visualization are saved in GitHub https://github.com/Ouyang2026/iMeta. Supplementary materials (figures, tables, graphical abstract, slides, videos, Chinese translated version, and update materials) may be found in the online DOI or iMeta Science http://www.imeta.science/.
