# Supplementary material for: Inhibition of ferroptosis by microbiota‐derived lithocholic acid underlies the intestinal radioprotection of a host defensin‐derived oligopeptide
Source: Imeta. 2026 Feb 19;5(1):e70113. doi: 10.1002/imt2.70113 (PMC13726622; doi:10.1002/imt2.70113)
Supplement: Supplementary file 1 [file IMT2-5-e70113-s001.docx]

**Supporting information to**

**Inhibition of ferroptosis by microbiota-derived lithocholic acid underlies the intestinal radioprotection of a host defensin-derived oligopeptide**

**Running title**: Defensin-derived oligopeptide alleviates ionizing radiation-induced intestinal injury

Xue Ouyang^1#^, Yingjuan He^1,2#^, Zihao Jin^1#^, Shaobo Wang^2#^, Gaomei Zhao^1^, Xiaona Su^3^, Jia Du^3^, Yin Chen^1^, Chengzhang Tan^1^, Xin Li^1^, Songling Han^1^, Bin Wang^4^, Jinghong Zhao^2^, Jianqin Niu^5,6^, Chuan Chen^3^*, Junping Wang^1^*, Cheng Wang^1^*

^1^State Key Laboratory of Trauma and Chemical Poisoning, Institute of Combined Injury of PLA, College of Preventive Medicine, Army Medical University, Chongqing 400038, China.

^2^Department of Nephrology, Xinqiao Hospital, Army Medical University, Chongqing 400037, China.

^3^Department of Cancer Center, Daping Hospital, Army Medical University, Chongqing 400042, China.

^4^Department of Gastroenterology, Chongqing Key Laboratory of Digestive Malignancies, Daping Hospital, Army Medical University, Chongqing 400042, China.

^5^Biomedical Analysis Center, Army Medical University, Chongqing 400038, China.

^6^Chongqing Key Laboratory of Neurobiology, Chongqing 400038, China.

^#^These authors contributed equally: Xue Ouyang, Yingjuan He, Zihao Jin, Shaobo Wang

*Correspondence: wangcheng@tmmu.edu.cn (Cheng Wang), wangjunping@tmmu.edu.cn (Junping Wang), sinkriver2012@tmmu.edu.cn (Chuan Chen)

**Figure S1** **Survival rates of female mice treated with HD5, AT9, or AT9(C/G) after TAI.** Mouse survival was monitored for 30 days post-TAI. Each group contains 10 mice.

**Figure S2** **Biocompatibility evaluation of a one-month course of AT9(C/G) in mice.** (A) Mouse body weight. (B) Blood cell counts. (C) Serum levels of alanine aminotransferase, (D) aspartate aminotransferase, (E) creatinine, and (F) urea. All quantitative data are presented as boxplots (*n* = 10 mice). ns, not significant. (G) Representative H&E images of the intestine, heart, liver, spleen, lung, and kidney from mice untreated or treated with AT9(C/G). Scale bar, 200 μm.

**Figure S3** **Localization of FITC-AT9(C/G) in *B. pseudolongum*.** Bacterial membranes are stained red (FM4‑64), while the peptide is labeled green (FITC). Bacterial DNA is stained with Hoechst 33342 (blue). Scale bar indicates 5 μm.

**Figure S4** **Viability of irradiated cells treated with different concentrations of bDCA or valerylcarnitine.** (A) Viability of irradiated IEC-6 and (B) HIEC-6 cells. Results are shown in the boxplot (*n* = 6 independent experiments). ^*^, *p* < 0.05; ^***^, *p* < 0.001.

**Figure S5** **Immunofluorescence characterization of human small intestinal organoids.** Human lysozyme and mucin 2 are immunostained in red, Ki67 is stained in green, and nuclei are probed with Hoechst 33342 (blue). Scale bar indicates 100 μm.

**Figure S6 Viability of irradiated HCT116 cells treated with or without LCA.** Results are shown in the boxplot (*n* = 6 independent experiments). ns, not significantly.

**Figure S7 Mitigative effect of LCA on IRIII in mice.** (A) H&E staining revealing the mitigative effect of LCA on IRIII in mice. Scale indicates 200 μm. (B) Villus length and number of crypts per millimeter in the ileum of mice. Results are shown in the boxplot (*n* = 6 mice). ^***^, *p* < 0.001.

**Figure S8 Serum LCA levels in irradiated mice with or without AT9(C/G) treatment.** Results are shown in the boxplot (*n* = 6 mice). ^**^, *p* < 0.01; ^***^, *p* < 0.001.

**Figure S9 The ratio of mean fluorescence intensity between oxidized and non‑oxidized probes.** Results are shown in the boxplot (*n* = 6 independent experiments). ^*^, *p* < 0.05; ^**^, *p* < 0.01.

**Figure S10 Representative images of HSIOs treated with erastin in the presence or absence of LCA.** Representative dead and survival HSIOs are indicated by red and blue arrows, respectively. Scale indicates 200 μm. Survival rates of HSIOs are shown as boxplots (*n* = 6 donors). ^***^, *p* < 0.001.

**Figure S11 LCA reprograms lipid metabolism in irradiated cells.** (A) Heatmap showing the effects of IR and LCA on levels of PA‑PLs, PC‑PLs, PG‑PLs, PI‑PLs, and PS‑PLs containing FA in IEC‑6 cells. Each group contains five independent samples. (B) Relative abundances of PA 38:5, PA 34:2, PC 42:7, PC 36:2, PG 36:4, PG 36:2, PI 36:4, PI 36:2, PS 38:4, PS 36:2, BMP 38:5, and BMP 36:2 in IEC‑6 cells. Results are shown in the boxplot. ^**^, *p* < 0.01; ^***^, *p* < 0.001.

**Figure S12 Top six functional domains identified through InterPro enrichment analysis.** The fatty acid desaturase was significantly enriched, with an enrichment ratio of 0.6 and a *p* value of 0.0004.

**Figure S13 Influence of LCA on the transcription of *Scd1*, *Fads1*, *Fads2*, *Gpx4*, *Nfe2l2*, and *Acsl4* in irradiated cells.** Results are shown in the boxplot (*n* = 6 independent experiments). ns, not significant; ^*^, *p* < 0.05; ^***^, *p* < 0.001.

**Figure S14 Assessment of siRNA-mediated gene knockdown by RT‑qPCR.** NC, negative control (HiPerFect transfection reagent). Target gene mRNA levels are shown as box plots (*n* = 3 independent experiments). The siRNAs selected for subsequent knockdown studies are marked with red arrows, and their sequences are provided in Table S5.

**Figure S15 Influence of OA supplementation on the adverse effects resulting from *Scd1* silencing on the anti-ferroptotic activity of LCA.** (A) Total Fe contents in the cells. Results are shown in the boxplot (*n* = 6 independent experiments). ^*^, *p* < 0.05. (B) MDA and (C) 4-HNE levels in the cells. Results are displayed as boxplots. ^***^, *p* < 0.001. (D) Viability of IEC-6 cells treated with 10 µM LCA and/or 12.5 µM OA, in the presence or absence of SCD1 knockdown. Results are shown in the boxplot. ^***^, *p* < 0.001.

**Figure S16 Immunofluorescence microscopy revealing the expressions of SCD1 and SREBP1 in IEC-6 cells.** Nuclei are stained by Hoechst 33342 (blue). The scale bar indicates 20 μm. Mean fluorescence intensities of SCD1 and SREBP1 in IEC-6 cells are shown in the boxplot (*n* = 6 independent experiments). ^***^, *p* < 0.001.

**Figure S17** **Maps of five truncated SCD1 reporter plasmids and a SREBP1 overexpression plasmid.** Shown are maps of pGL3-SCD1-P1, pGL3-SCD1-P2, pGL3-SCD1-P3, pGL3-SCD1-P4, pGL3-SCD1-P5, pGL3-SCD1-MUT, and SREBP1 overexpression plasmids.

**Figure S18 Ratios of p‑AKT to AKT in mouse ileum following LCA or AT9(C/G) treatment.** (A) Ratio of p‑AKT to AKT in the ileum of mice treated with LCA or (B) AT9(C/G). Results are shown in the boxplot (*n* = 4 mice). ^**^, *p* < 0.01; ^***^, *p* < 0.001.

**Figure S19 Villus length and number of crypts per millimeter in the ileum of irradiated mice.** Results are shown in the boxplot (*n* = 6 mice). ^***^, *p* < 0.001.

**Figure S20 HPLC and MS spectra of synthetic peptides.** Peptide purities are all above 90%. The observed molecular weights of these peptides are close to their theoretical values.

**Figure S21 Genotyping results of *Gpbar1*^-/-^ mice.** (A) Strategy of genotyping. (B) Primer information. (C) Gel image. Homozygotes show a single band at 374 bp, while wild-type mice exhibit a single band at 536 bp.
